# Analysis of damage-associated molecular patterns in amyotrophic lateral sclerosis based on ScRNA-seq and bulk RNA-seq data

**DOI:** 10.3389/fnins.2023.1259742

**Published:** 2023-10-24

**Authors:** Yue Shi, Ruixia Zhu

**Affiliations:** Department of Neurology, The First Affiliated Hospital of China Medical University, Shenyang, China

**Keywords:** DAMPs, ALS, single-cell sequencing, molecular docking, immune infiltration

## Abstract

**Background:**

Amyotrophic Lateral Sclerosis (ALS) is a devastating neurodegenerative disorder characterized by the progressive loss of motor neurons. Despite extensive research, the exact etiology of ALS remains elusive. Emerging evidence highlights the critical role of the immune system in ALS pathogenesis and progression. Damage-Associated Molecular Patterns (DAMPs) are endogenous molecules released by stressed or damaged cells, acting as danger signals and activating immune responses. However, their specific involvement in ALS remains unclear.

**Methods:**

We obtained single-cell RNA sequencing (scRNA-seq) data of ALS from the primary motor cortex in the Gene Expression Omnibus (GEO) database. To better understand genes associated with DAMPs, we performed analyses on cell–cell communication and trajectory. The abundance of immune-infiltrating cells was assessed using the single-sample Gene Set Enrichment Analysis (ssGSEA) method. We performed univariate Cox analysis to construct the risk model and utilized the least absolute shrinkage and selection operator (LASSO) analysis. Finally, we identified potential small molecule drugs targeting ALS by screening the Connectivity Map database (CMap) and confirmed their potential through molecular docking analysis.

**Results:**

Our study annotated 10 cell types, with the expression of genes related to DAMPs predominantly observed in microglia. Analysis of intercellular communication revealed 12 ligand-receptor pairs in the pathways associated with DAMPs, where microglial cells acted as ligands. Among these pairs, the SPP1-CD44 pair demonstrated the greatest contribution. Furthermore, trajectory analysis demonstrated distinct differentiation fates of different microglial states. Additionally, we constructed a risk model incorporating four genes (TRPM2, ROCK1, HSP90AA1, and HSPA4). The validity of the risk model was supported by multivariate analysis. Moreover, external validation from dataset GSE112681 confirmed the predictive power of the model, which yielded consistent results with datasets GSE112676 and GSE112680. Lastly, the molecular docking analysis suggested that five compounds, namely mead-acid, nifedipine, nifekalant, androstenol, and hydrastine, hold promise as potential candidates for the treatment of ALS.

**Conclusion:**

Taken together, our study demonstrated that DAMP entities were predominantly observed in microglial cells within the context of ALS. The utilization of a prognostic risk model can accurately predict ALS patient survival. Additionally, genes related to DAMPs may present viable drug targets for ALS therapy.

## 1. Introduction

The neurodegenerative disease amyotrophic lateral sclerosis (ALS) affects motor neurons in the upper and lower limbs. Presently, there is no effective treatment for this condition. The global prevalence rate of ALS is 4.42 per 100,000 individuals, with an escalating incidence with advancing age (Ingre et al., 2015; Xu et al., 2020). ALS can impact the muscles in the spinal cord and the bulbar area, in addition to the respiratory system (Brown and Al-Chalabi, 2017). The clinical heterogeneity of ALS poses a challenge for diagnosis, and the etiology remains unclear, particularly in the absence of diagnostic tests (Bordoni et al., 2020). Moreover, proof is scarce regarding intervention strategies for ALS. Despite being approved by the FDA, Rilutek and Radicava have demonstrated limited effectiveness in managing ALS symptoms (Jaiswal, 2019). Moreover, the majority of patients succumb to the disease within 3 to 4 years of exhibiting symptoms (van Es et al., 2017). Thus, it is crucial to gain a comprehensive comprehension of ALS.

Damage-Associated Molecular Patterns (DAMPs) encompass a collection of molecules capable of initiating and perpetuating immune responses within non-infectious inflammatory contexts (Thundyil and Lim, 2015). Typically sequestered within cellular confines, these molecules are exclusively released into the extracellular milieu after cellular damage or exposure to stressors. In this extracellular domain, they assume a pivotal role as potent activators of the immune system. DAMPs exhibit an affinity for diverse receptors, encompassing toll-like receptors (TLRs) and the receptor for advanced glycation end products (RAGE), thereby eliciting intricate signaling cascades that culminate in inflammatory and immune responses (Cruickshank et al., 2018; Roh and Sohn, 2018; Vaes et al., 2021). Hence, the understanding and comprehensive knowledge of DAMPs’ functionality assumes paramount importance in delineating the pathogenic underpinnings of an array of diseases, encompassing but not limited to cancer and neurodegenerative disorders. In tumors, DAMPs are widely regarded as one of the most auspicious approaches for eradicating tumor cells. The primary role of DAMPs is to activate the immune system’s reaction to cancer, which leads to the mobilization of anti-tumor cells, the secretion of anti-cancer cytokines, and the suppression of tumor development (Ashrafizadeh et al., 2020). In a multitude of cancer types, various immune cell populations are activated in response to DAMPs. Illustrative instances from diverse categories of cancer serve to underscore this phenomenon. For instance, Myeloid-derived suppressor cells (MDSCs) are activated upon encountering DAMPs in breast cancer (Vrakas et al., 2015). In the context of Lung Cancer: DAMPs activate dendritic cells, prompting an immune response that frequently contributes to inflammatory conditions during the progression of the tumor (Solari et al., 2020). In the context of Pancreatic Cancer: Tumor-associated macrophages (TAMs) are recognized for their activation in reaction to DAMPs (Naqvi et al., 2018). Notably, in the context of ALS, various studies have indicated an increase in specific DAMPs. For instance, the levels of Toll-like receptor 4 (TLR4), a prominent DAMP, were found to be significantly elevated in the activated microglia of sporadic ALS cases (Casula et al., 2011). Moreover, the interaction between misfolded wild-type SOD1, a protein often mutated in familial ALS, and TLR4, triggers neuroinflammatory processes resulting in neurotoxic effects (Lee et al., 2015). Hence, it indicates a potential therapeutic target for slowing the progression of ALS, with treatments aimed at preventing DAMP formation or inhibiting their receptor activation.

This study primarily elucidated the involvement of DAMPs in ALS through two distinct perspectives. Firstly, at the single-cell level, we identified the presence of DAMPs-inducing conditions in ALS. Furthermore, we developed a novel and validated prognostic model for ALS at the bulk RNA-seq level, examining the clinical significance and immune cell infiltration status of both high and low-risk cohorts. Additionally, we utilized molecular docking technology to anticipate potential drug candidates, thereby furnishing a theoretical foundation for the advancement of ALS medication. Lastly, we investigated the potential use of these genes as drugs of choice.

## 2. Methods

### 2.1. Single-cell RNA sequencing data processing

**Figure 1:**
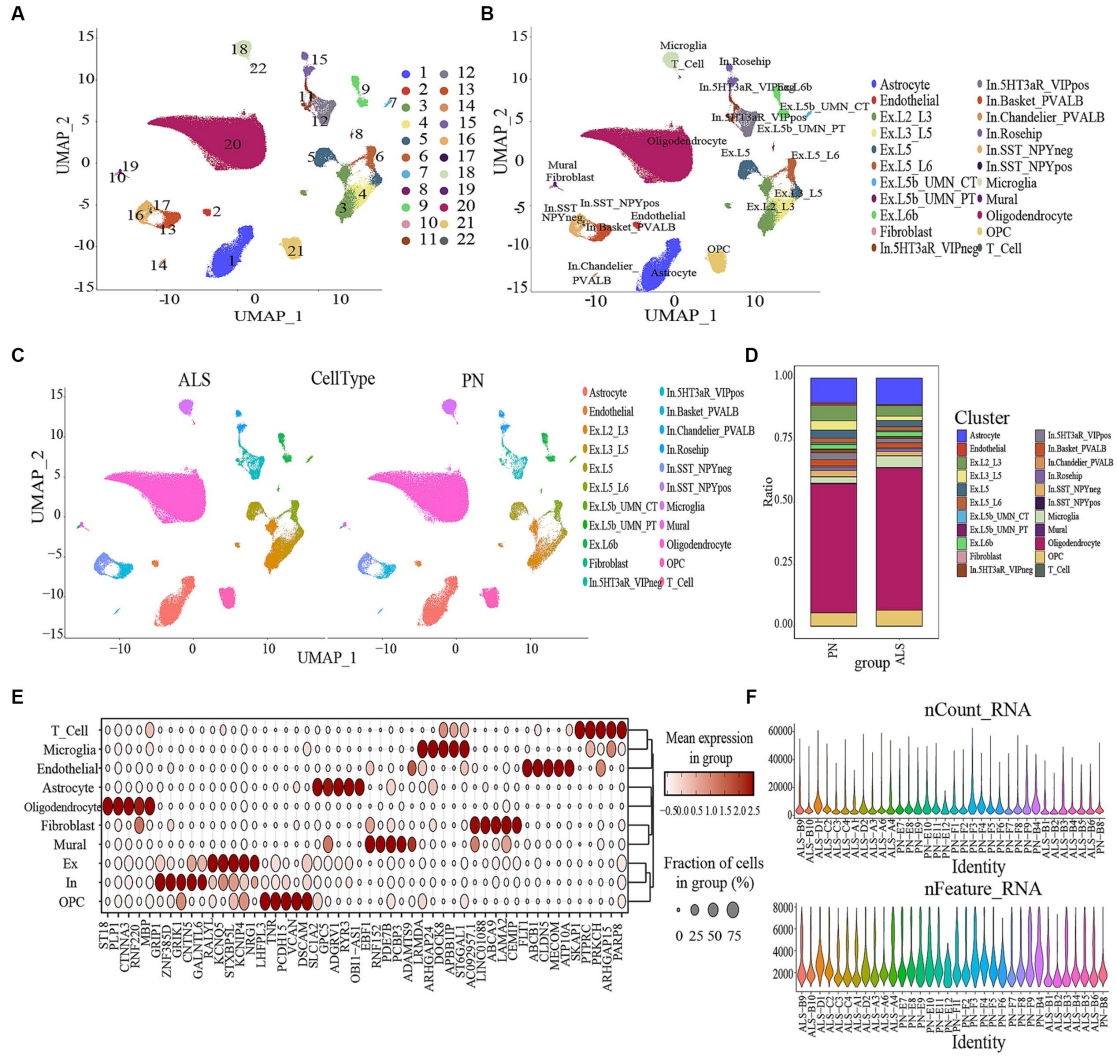
Data processing and defining cell types. **(A)** Umap of the 22 cell clusters. **(B)** The cell types were identified by marker genes. Abbreviations: Ex, Excitatory; In, Inhibitory **(C)** The expression of cell types in ALS and PN. **(D)** Proportion of cells in ALS and PN. **(E)** Expression levels of top 5 marker genes for each cell type. **(F)** Gene data detected in each cell, the total number of molecules detected in the cell; the X-axis and color represent different sample numbers, and the Y-axis represents the number of detected genes.

The study utilized GSE174332, which consisted of 17 cases with ALS and 17 pathologically normal controls (PN) with comparable sex distributions, for RNA sequencing analysis of the primary motor cortex (Pineda et al., 2021). To ensure the selection of single cells, the DoubletFinder package, version 2.0.3, was employed and specific criteria were applied, including nFeature RNA > 200, percent mitochondria>20%, and nCount RNA > 1,000, to eliminate doublets and deceased cells. Cells that did not meet the criteria of having at least 6,000 genes, a total of more than 200 genes, or mitochondrial genes greater than 20% were excluded from the analysis (Figure 1F). The gene expression data was standardized and adjusted using the “Lognormalizer” technique.

**Figure 2:**
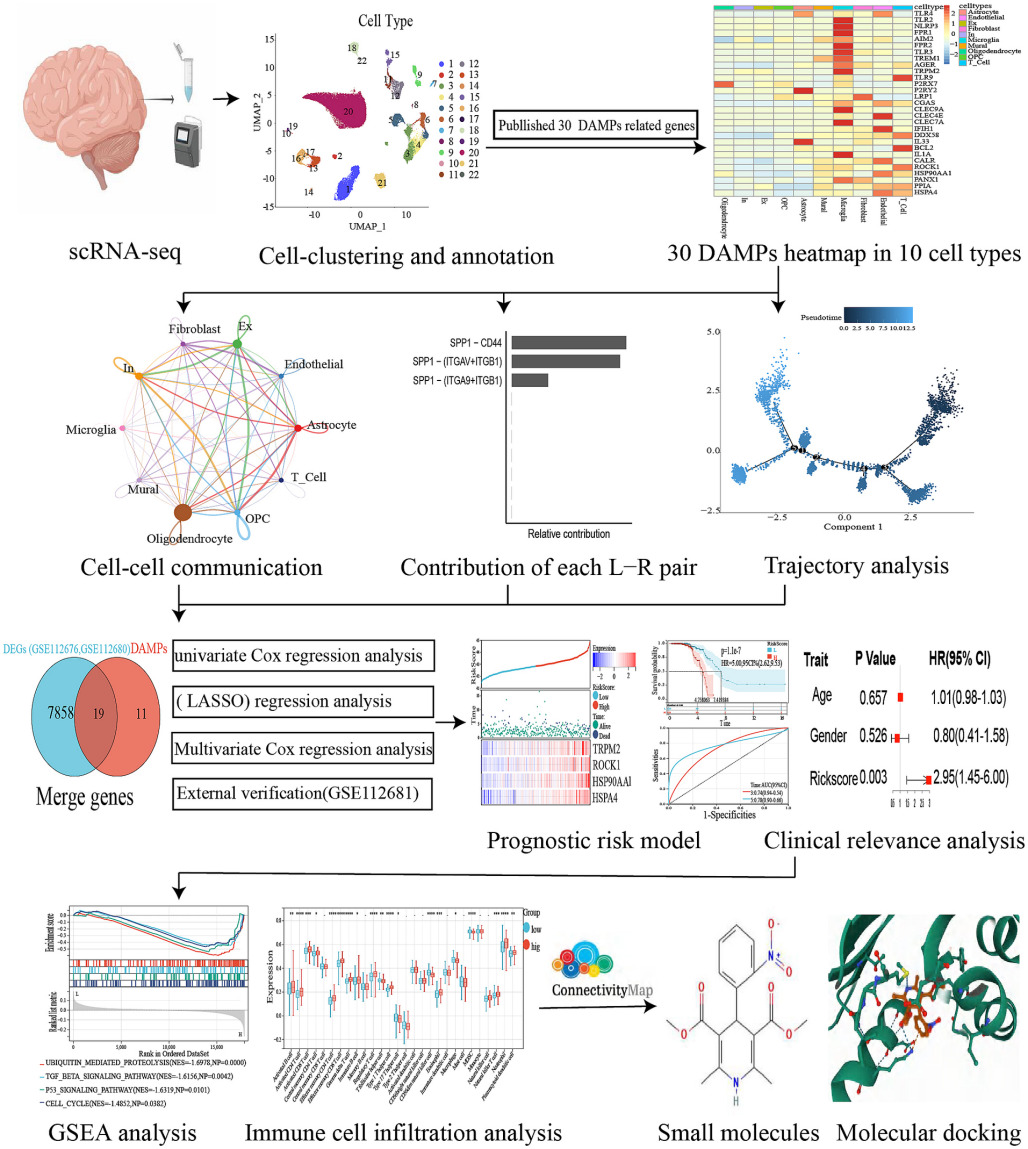
Flowchart of our study.

To differentiate each sample, a set of 3,000 genes with high variability (HVGs) was employed, using a “vst” approach and a “harmony” R package for batch correction. The application of “FindNeighbors,” “FindClusters,” and “runUMAP” functions from Seurat produced a 2D map that displayed clusters that had undergone a dimensionality reduction (Hao et al., 2021). The automation of relevant cell-type annotation within this structure was facilitated through the utilization of the ACTIONet R package. The runACTIONet function was implemented with a depth set to 30, followed by the application of the annotate.cells.using.markers function for cell annotation. The resulting annotations were integrated into the Seurat object. The classification of cells into 26 distinct clusters was carried out using FindClusters with a resolution of 0.3. These clusters were subsequently categorized into 22 cell types using the ACTIONet R package. The veracity of these classifications was validated via manual examination of the output from the “FindAllMarkers” algorithm. The algorithm “FindAllMarkers” was employed to filter genes with a logfc of 0.25, which was considered to indicate minimal disparities (Sinha et al., 2018). Afterward, the markers underwent filtration by applying a revised *p*-value thresholdof below 0.05. To evaluate the difference in gene expression levels between groups, the Wilcoxon rank sum test was employed. A heatmap was constructed using 30 selected DAMPs based on identifying 10 cell types. The flowchart of our study was shown in Figure 2.

### 2.2. Go, KEGG, and GSEA

A GSEA analysis was conducted to detect genes exhibiting significant differential expression across various cell groups. To perform GO and KEGG enrichment analysis on the differentially expressed genes of microglia and OPC (log2FC > 0.25, adj *p* value <0.05), the GSE174332 dataset was utilized. Subsequently, the enrichment outcomes were compared between the two cell populations.

### 2.3. Cellchat in amyotrophic lateral sclerosis

**Figure 3:**
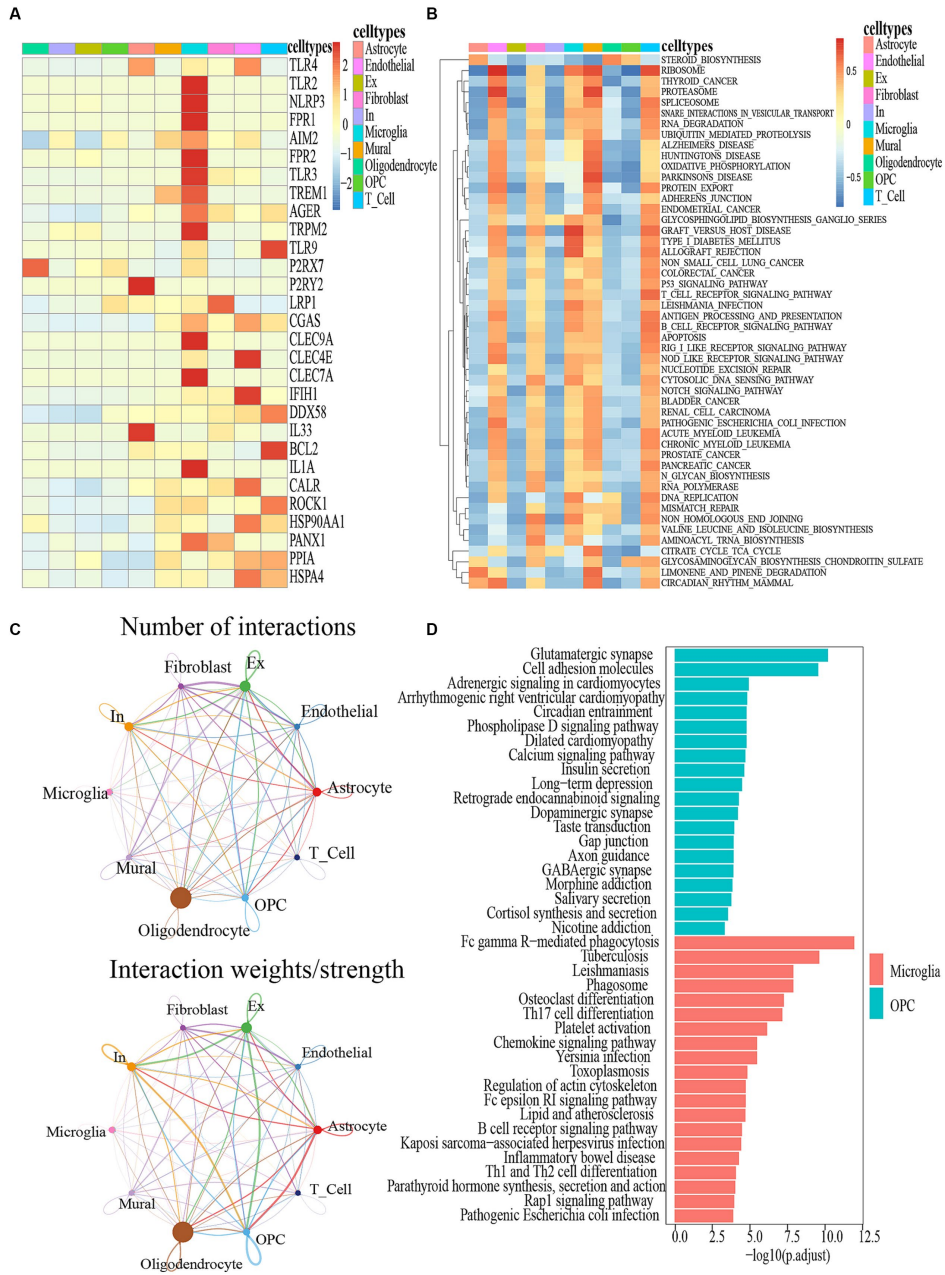
**(A)** DAMPS marker gene heatmap in 10 cell types. **(B)** GSEA for differentially expressed genes between different cell types. **(C)** Circle plot showing the intercellular communication and the interaction strength between major cell types in ALS, colored according to each cell type; the thickness degree indicates the interaction strength between sender and receiver cell. **(D)** KEGG analysis of the differentially expressed genes between the Microglia and OPC clusters.

**Figure 4:**
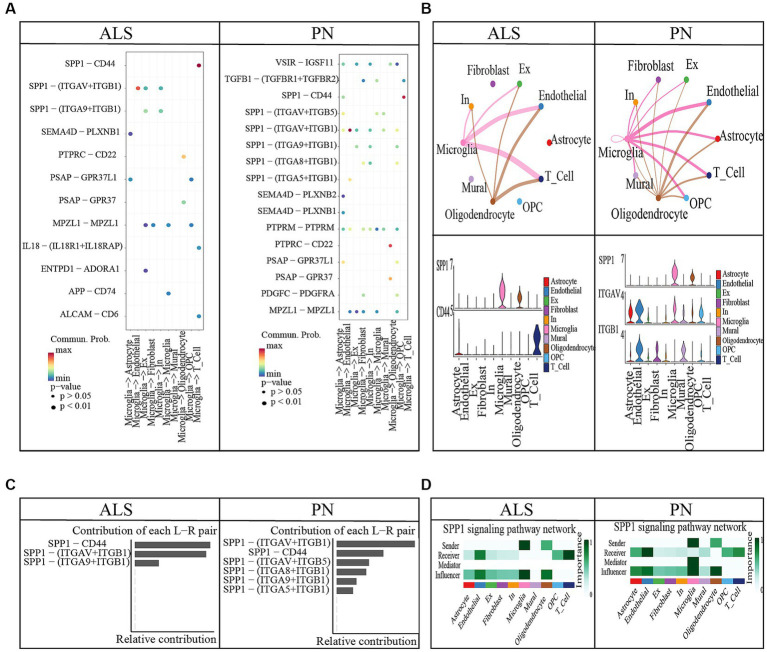
Cell–cell communication between ALS and PN. **(A)** Microglia release ligands that act on 10 cells. **(B)** Circle plots illustrated and compared alterations in cell–cell communication between ALS and PN; SPP1-CD44 and SPP1 − (ITGAV+ITGB1) expression in 10 cell types. **(C)** Contribution of each L − R pair. **(D)** SPP1 signaling pathway network.

Firstly, the CellChat and patchwork packages were utilized for generating CellChat objects, establishing ligand-receptor interaction databases, and preprocessing expression data. Secondly, to calculate the CellChat network, we used the “trimean” function to estimate communication probability. Data frames were then extracted from the CellChat network, signaling pathways were used to determine communication, and cellular communication networks were computationally integrated (Figure 3C). Thirdly, signaling pathways associated with DAMPs were visualized. Each ligand-receptor pair was calculated according to its contribution to each pathway, and the regulatory role of ligand-receptor pairs in cellular communication was shown in Figure 4.

### 2.4. Trajectory analysis of single cells

The cellular state undergoes continuous fluctuations throughout the course of development (Haghverdi et al., 2016; Sun et al., 2022). Microglial subsets undergo analysis utilizing a pseudo-time sequence, whereby markers distinctive to these subsets were utilized for cell categorization (Supplementary Table S3). This methodology facilitated the assessment of the overarching interplay between alterations in cellular expression. The R package Monocle2 was used to generate single-cell pseudotime trajectory plots (Trapnell et al., 2014). This R package diminished the high-dimensional expression profile into lower-dimensional space using a machine learning technique (Qiu et al., 2017). An object was created using the “newCellDataSet” function, with the parameter expression family set to neg binomial size. Only genes with an average expression of at least 0.1 were taken into account for the trajectory analysis. The function “reduce dimension” was utilized to perform dimension reduction, taking DEGs between the cell groups as input and setting the parameters method to “DDRTree” and max components to 2. The function “plot cell trajectory” was employed to organize and examine cells. The function “plot genes branched heatmap” was used to classify and visualize the genes identified using the branch expression analysis modeling (BEAM) analysis (Zhang et al., 2021).

### 2.5. Acquisition of datasets and damage-associated molecular pattern-related genes

The GEO database, maintained by NCBI, provides extensive gene expression information using microarray and sequence-based methods (Barrett et al., 2013). The present study included two datasets, namely GSE112676 and GSE112680. A total of 233 individuals diagnosed with ALS and 508 control subjects were examined in the study GSE112676. The dataset GSE112680, created with the use of Illumina HumanHT-12 Expression BeadChip (GPL10558), consisted of 164 individuals diagnosed with ALS and 137 individuals serving as controls. The Illumina platform served as the basis for the GSE112676 and GSE112680 datasets, which contain identical clinicopathological information on ALS patients. Included in this data are the time when symptoms first appeared (known as age at onset), the affected area (either the bulbar region or the spinal region), the individual’s gender (male or female), the duration of follow-up, and the current condition (whether the patient is alive or deceased).

Moreover, the merging of the datasets was accomplished by employing the R package called inSilicoMerging (Taminau et al., 2012). Subsequently, the methodology by Johnson WE et al. was employed to eliminate any potential batch effects (Johnson et al., 2006). In the two microarray datasets, the “ComBat” algorithm was applied to mitigate batch effects (Leek et al., 2012). To showcase the effectiveness of the normalization and batch correction techniques, UMAP analysis was performed on the microarray data both before and after applying these methods (Supplementary Figure S1). After removing the batch effect of GSE112676 and GSE112680, we identified 7,877 DEGs between ALS and control samples, which included 4,100 upregulated and 3,777 downregulated genes (Supplementary Table S1).

Supplementary Table S2 presents the information on 30 genes associated with DAMPs, which were gathered based on prior research (Hernandez et al., 2016; Galluzzi et al., 2020; Gong et al., 2020).

### 2.6. Construction of the prognostic model

As a first step, a Cox regression analysis was conducted to identify potential prognostic DEGs (van Dijk et al., 2008). LASSO was used to perform regression analysis, with variables with *p*-values below 0.05 selected for regression analysis (Tibshirani, 1997). To reduce the gene count and avoid overfitting, the LASSO regression analysis was applied in the final risk model using the R software “glmnet” package. Afterward, the genes discovered via LASSO regression were analyzed using multivariate Cox regression, leading to the creation of a prognostic model using the subsequent equation: Risk scores = sum of (coefficient × expression of a signature gene; Christensen, 1987). Based on the median risk score value, the patients were divided into high-risk and low-risk cohorts. To depict the discrepancy in survival and the condition of every individual, we utilized the R software’s “survminer” and “ggrisk” packages to create survival curves and risk plots. Additionally, ROC curves were generated using the R software’s “timeROC” package, evaluating the predictive capacity of the risk score for the overall survival (OS) of ALS patients over 1, 3, and 5 years.

### 2.7. Examining the clinical significance and enrichment of high-risk and low-risk groups

The potential of the risk score as an independent prognostic factor for ALS patients was investigated through Cox regression analysis using the R software, particularly the “survival” package. Furthermore, the forest plot package was employed to produce forest plots for both univariate and multivariate Cox regression analyses. Additionally, Cox proportional hazards regression was utilized to conduct univariate and multivariate logistic analyses, evaluating the prognostic significance of the risk score in combination with other clinical factors and the prognosis-related gene signature. We determined the hazard ratios (HR) and calculated the corresponding 95% confidence intervals (CI). To identify the pathways with the greatest level of enrichment between the high- and low-risk groups, the R software packages “clusterProfiler” and “enrich plot” were utilized for the Gene Set Enrichment Analysis (GSEA).

### 2.8. Immune infiltration

To assess the level of immune cell enrichment, earlier studies utilized gene sets, while ssGSEA (single-sample gene set enrichment analysis) was employed for determining infiltrating immune cells (Hänzelmann et al., 2013; Charoentong et al., 2017). The distribution of immune cell types is characterized by a relative abundance, with 0 indicating the lowest and 1 indicating the highest. Additionally, the Wilcoxon test was used to compare samples obtained from individuals with ALS and controls.

### 2.9. Small molecule drug analysis and molecular docking

To predict small-molecule drugs targeted the ALS and control DEGs, the Connectivity Map (CMAP) from https://clue.io/ was employed. The connectivity score (median tau score) was computed to establish ranking and filtering, with a range of 100 to −100. A positive connectivity score of a compound results in comparable changes to the uploaded genes, while a negative connectivity score of a compound leads to opposite changes to the uploaded genes, indicating its potential as a promising drug. Perturbagens with connectivity scores below 95 were deemed significant candidates.

The drug candidate and its targets were analyzed for binding affinities and interaction modes using AutodockVina 1.2.2, a software for protein-ligand docking simulations (Morris et al., 2008). Low binding energies between ligand and receptor result in a more stable conformation. The molecular structures of potential drugs were acquired from PubChem Compound (Wang et al., 2017).^1^ Meanwhile, the 3D coordinates of TRPM2 (PDB ID, 7AOV; resolution, 2.00 Å), ROCK1 (PDB ID, 5WNE; resolution, 2.60 Å), HSP90AA1 (PDB ID, 4BQG; resolution, 1.90 Å), and HSPA4 (PDB ID, 3GLA; resolution, 1.64 Å) were retrieved from the PDB.^2^ To perform docking analysis, the protein and molecular files were converted to PDBQT format, with the exclusion of water molecules and the inclusion of polar hydrogen atoms. To enable unrestricted molecular movement, the domain of each protein was encompassed by a centered grid box. The size of the grid box was set to 30 angstroms in length, width, and height, with a grid point spacing of 0.05 nanometers. The molecular docking studies were carried out using Autodock Vina 1.2.2.^3^

### 2.10. Statistical analysis

The present study utilized R Statistical Software (version 4.1.2) to analyze distinctions among the groups using Wilcox tests. Predictive models were developed using LASSO regression and Cox regression analyses. Using the Kaplan–Meier method, survival analysis was performed, and log-rank tests were used to assess differences between the groups. *p* < 0.05 defines statistical significance.

## 3. Results

### 3.1. Single-cell profiling in amyotrophic lateral sclerosis

The ScRNA sequencing dataset (GSE174332) comprises a total of 199,556 cells, with 121,224 cells derived from ALS and 78,332 cells derived from pathologically normal controls (PN). A total of 176,253 cells were retained through the filtration process, consisting of 109,196 cells from ALS and 67,057 cells from PN. The cells were categorized into 22 different clusters (Figure 1A). Based on marker genes, the clusters were further classified into various cell types (Figures 1B,C). Subpopulation analysis revealed seven excitatory and inhibitory neuron subtypes (Ex Neuron and In Neuron), respectively (Figure 1B). Figure 1D showed the different ratio of cell types between ALS and PN. Supplementary Figure S5 depicted the expression patterns of the top 5 marker genes across the 10 cell types. To gain a deeper comprehension of the attributes of every cell subcategory, we examined the marker genes linked to each subcategory. Figure 1E showed the violin plot illustrating the top five genes across all subpopulations. Our analysis identified 10 cell types, namely OPC (Oligodendrocyte precursor cell), Ex Neuron (Excitatory Neuron), In Neuron (Inhibitory Neuron), Mural, Fibroblast, Oligodendrocyte, Astrocyte, Endothelial, Microglia, and T cells.

The expression of DAMPs-related genes was primarily observed in microglia (Figure 3A). Figure 3B depicted the top 50 pathways of the utmost significance of GSEA analysis, categorized as immunity-related, biosynthesis-related, apoptosis-related, etc. It was widely acknowledged that the manifestation of ALS was intricately linked to the aberrant expression of these pathways. Firstly, the TGF-β Signaling Pathway, an immunity-related pathway, is instrumental in modulating glial cell activation and inflammatory responses. Activated glial cells release an array of cytokines, thereby instigating inflammation and neurodegeneration (Si et al., 2015; Tripathi et al., 2017). Secondly, Steroid biosynthesis, a biosynthesis-related pathway, especially glucocorticoid biosynthesis, has exhibited anti-inflammatory and neuroprotective effects that could potentially confer benefits for individuals with ALS (McLeod et al., 2020). Furthermore, the engagement of glial cells in the steroid biosynthesis pathway becomes evident through their active role in generating essential steroid intermediates and metabolites (Manna et al., 2015). Thirdly, the P53 signaling pathway, a pathway closely linked to apoptosis, is primarily relevant to motor neurons. Renowned for its pivotal role in regulating apoptosis, this pathway has also exhibited intricate connections with ALS (Ranganathan and Bowser, 2010). Furthermore, The GSEA analysis revealed a notable concurrence between the top 50 pathways and the KEGG enrichment pathways associated with DAMPs-related genes (Supplementary Figure S6). Particularly, the steroid biosynthesis pathway exhibited the highest significance by ranking first among these shared pathways (Figure 3B). In comparison of genes expressed by microglial cells and OPCs, KEGG analysis revealed that 20 pathways were enriched in each group (Figure 3D). Furthermore, a comprehensive KEGG analysis of other cell types has been visually presented in Supplementary Figure S7. Hence, the KEGG enrichment results revealed a strong association between the expression of genes related to microglial cells and pathways associated with DAMPs.

### 3.2. The amyotrophic lateral sclerosis cell chat results

ALS samples exhibited 12 unique ligand-receptor pairs within DAMP-related pathways, involving microglial cells as ligands when compared to PN. These pairs included APP-CD74, ENTPD1-ADORA1, IL18-(IL18R1 + IL18RAP), MPZL1-MPZL1, PSAP-GPR37, PSAP-GPR37L1, PTPRC-CD22, SEMA4D-PLXNB1, SPP1-(ITGA9 + ITGB1), SPP1-(ITGAV+ITGB1), and SPP1-CD44 (Figure 4A). Among these pairs, the SPP1-CD44 ligand-receptor pair showed the highest level of contribution in ALS, while the SPP1-(ITGAV+ITGB1) ligand-receptor pair exhibited the highest level of contribution in PN (Figure 4C). In the comparison between ALS and PN cells (Figure 4B), the majority of signals displayed a general decrease in ALS compared to PN. In ALS, the center was typically localized in microglial cells. However, this microglial-cell-centered pattern was disrupted as endothelial cells, astrocytes, T cells, and OPCs increasingly became involved in PN (Figure 4B). Moreover, the hierarchical diagrams depicted the autocrine and paracrine communication of cells within the signaling pathway. In the diagrams, solid circles represented the origin of the communication, while hollow circles indicated the destination. Consequently, it was observed that microglial cells served as the primary sources of communication in both ALS and PN (Figure 4D).

### 3.3. Trajectory results of single cells

**Figure 5:**
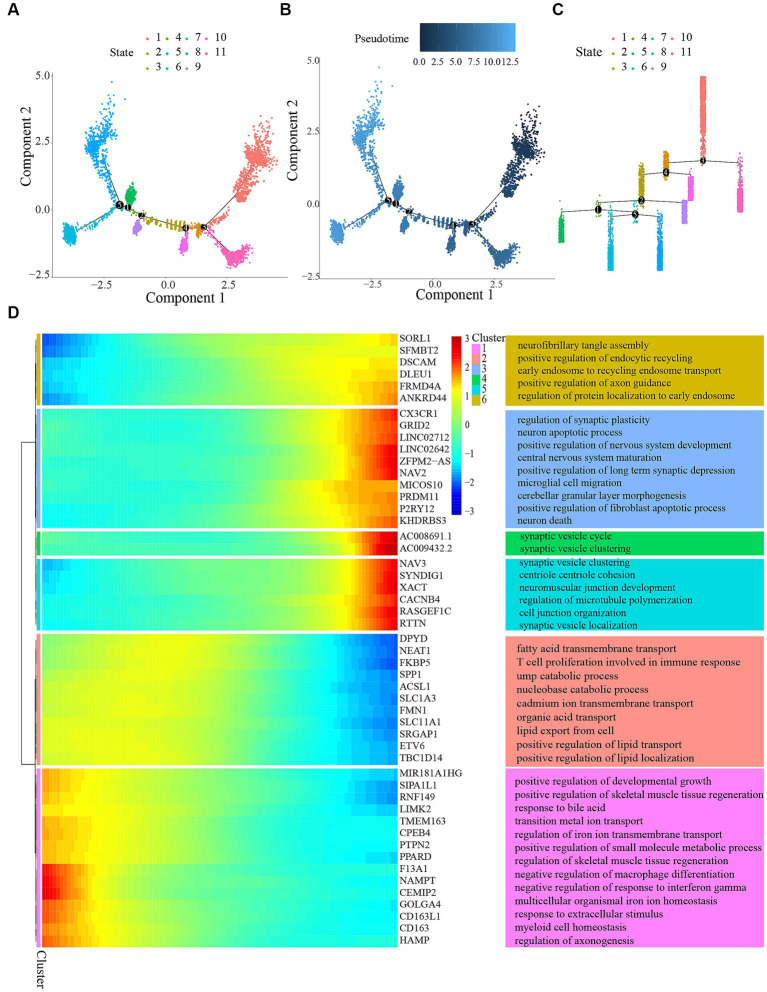
Trajectory analysis of microglial cells. **(A)** Pseudotemporal trajectory of microglia cells subclusters using the Monocle algorithm. **(B)** Microglia cells evolved from dark blue to light blue. Darker blue represents an earlier stage of differentiation, while lighter blue indicates a later stage of differentiation. **(C)** Distribution of different states in microglia. **(D)** Differentially expressed genes along the pseudotime were hierarchically clustered into six subclusters. The top annotated GO terms in each cluster were provided.

Using Monocle software, we performed a pseudo-time series analysis to confirm the development stages of different microglial groups. The results suggested that the four groups can be broadly classified into five distinct states of differentiation (Figures 5A,C). Figure 5B symbolized the temporal dimension of cellular differentiation. Developmental stages occur earlier as the color becomes darker. The findings indicated that cells in Cluster 1was in the initial phase of growth, whereas cells in Cluster 8 were in the final phase of development (Figure 5B).

To determine genes controlled in a manner specific to a branch, we created a heatmap (Figure 5D). The heatmap’s columns symbolize nearly consecutive points, whereas the genes were symbolized by the rows. When reading from the center of the heatmap toward the right, a lineage of quasi-time series becomes apparent, while reading toward the left uncovers a different lineage. The analysis revealed alterations in genes that were expressed differently and carried out various gene expression programs. The genes were categorized into six clusters associated with the promotion of skeletal muscle tissue regeneration, assembly of neurofibrillary tangles, migration of microglial cells, neuronal demise, development of neuromuscular junctions, and more.

### 3.4. Prognostic model construction and validation

**Figure 6:**
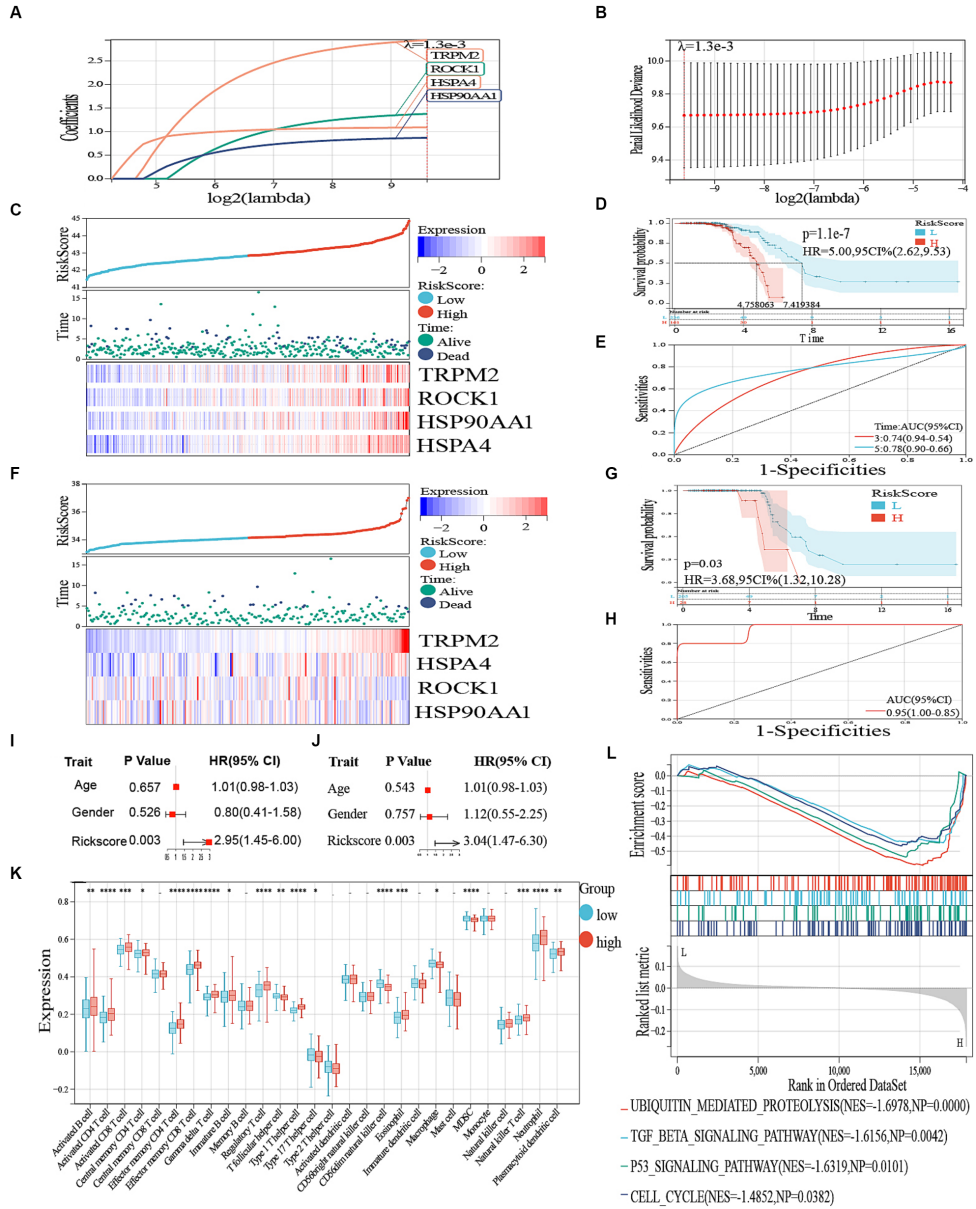
**(A)** LASSO regression of the 19 genes. **(B)** Cross-validation for optimizing the parameter in LASSO regression. **(C)** The distribution of risk score and survival status and a heatmap generated based on identified gene expression. **(D)** Kaplan–Meier curves of survival analysis. **(E)** ROC analysis for predicting the risk of death. **(F)** The distribution of risk score and survival status in GSE112681 and a heatmap generated based on identified gene expression. **(G)** Kaplan–Meier curves of survival analysis in ALS. **(H)** ROC analysis for predicting the risk of death in GSE112681. **(I)** Univariate Cox regression analysis revealed the association between patients’ survival and clinicopathological parameters. **(J)** Multivariate Cox regression analysis uncovered that only the risk score (*p* < 0.05) was an independent prognostic factor for ALS patients. **(K)** Boxplot of high and low risk between different immune cells. – represents nonsense, * represents *p* < 0.05, ** represents *p* < 0.01; *** represents *p* < 0.001. **(L)** GSEA to investigate the biological processes and pathways enriched in high- and low risk groups.

To identify potential prognostic DAMPs-related DEGs in ALS, we conducted a univariate Cox regression analysis. After intersecting 7,877 DEGs and 30 DAMPs-related genes, we obtained 19 DAMPs-related DEGs, including TLR4, TLR2, NLRP3, FPR1, AIM2, FPR2, TREM1, TRPM2, TLR9, P2RX7, P2RY2, CGAS, CLEC4E, CLEC7A, IFIH1, CALR, ROCK1, HSP90AA1, and HSPA4. In total, four genes were identified as prognostic DEGs (*p* < 0.05; Supplementary Figure S2). Afterward, we conducted a LASSO regression analysis to decrease the amount of DEGs in the ultimate risk model. Through this step, we identified four genes (Figures 6A,B). Finally, using multivariate Cox analysis, we recognized four genes as independent prognostic DEGs. These genes included TRPM2, ROCK1, HSP90AA1, and HSPA4. According to the survival curve, patients classified as high-risk had a lower overall survival (OS) rate compared to patients classified as low-risk (Figures 6B–D). Furthermore, the risk score exhibited excellent predictive accuracy for overall survival in these subjects, with an area under the curve (AUC) of 0.74 and 0.78 for 3-year and 5-year overall survival, respectively (Figure 6E). Comparable findings were noted in the GSE112681 dataset (Figures 6B,F,G). Figures were created to demonstrate the specific survival results of individual patients in the external validation groups (AUC = 0.95; Figure 6H). For this investigation, we employed the R program glmnet to combine information on survival time, survival status, and gene expression data. Additionally, we utilized the lasso-cox technique for conducting regression analysis. Furthermore, we established a 10-fold cross-validation technique to acquire the most suitable model. After setting the Lambda value to 0.00127609072719019, we ultimately acquired a total of four genes. The constructed model formula is RiskScore = 2.94049083002057*TRPM2 + 1.3768606558898*ROCK1 + 0.868830633563441*HSP90AA1 + 1.08881045581752*HSPA4. The formulated model equation was incorporated into the individual cell data. The dot plot in Supplementary Figure S3 depicted the expression of four prognostic genes in the single-cell data. The scatter plot in Supplementary Figure S4 exhibited the dynamic manifestation of these four predictive genes with pseudo-temporal values.

### 3.5. Clinical significance and immune cell infiltration

We then performed a single-variable and multivariate Cox analysis on the risk score to determine if it could act as a standalone prognostic factor. According to Figures 6I,J, the univariate Cox regression analysis showed that there was a favorable correlation between solely the risk scores and the overall survival (HR 2.95, 95% CI 1.45–6.00, *p* = 0.003). Moreover, the multivariate analysis revealed a significant association between the prognostic risk score (HR 3.04, 95% CI 1.47–6.30, *p* = 0.003) and the overall survival of individuals with ALS, indicating its potential as a standalone prognostic indicator. Moreover, we performed a GSEA to detect the pathways that exhibited significant enrichment. The results of our analysis showed significant enrichment of genes in the TGF beta signaling pathway, ubiquitin-mediated proteolysis, p53 signaling pathway, and cell cycle (Figure 6L).

An inquiry was carried out to ascertain the association between immune-related genes and ALS by examining the infiltration of immune cells. A total of 28 immune cells infiltrating ALS were screened with values of *p* < 0.05, as shown in Figure 6K. The findings indicated that the high-risk group exhibited increased expression of activated B cells, activated CD8 and CD4 T cells, effector memory CD4 T and CD8 T cells, Gamma delta T cells, immature B cells, Type 1 T helper cells, eosinophils, regulatory T cells, and plasmacytoid dendritic cells (*p* < 0.05). Conversely, the low-risk group demonstrated higher expression of central memory CD4 T cells, macrophages, MDSC (Myeloid-derived suppressor cells), natural killer T cells, and neutrophils (Figure 6K).

### 3.6. Molecular docking

**Figure 7:**
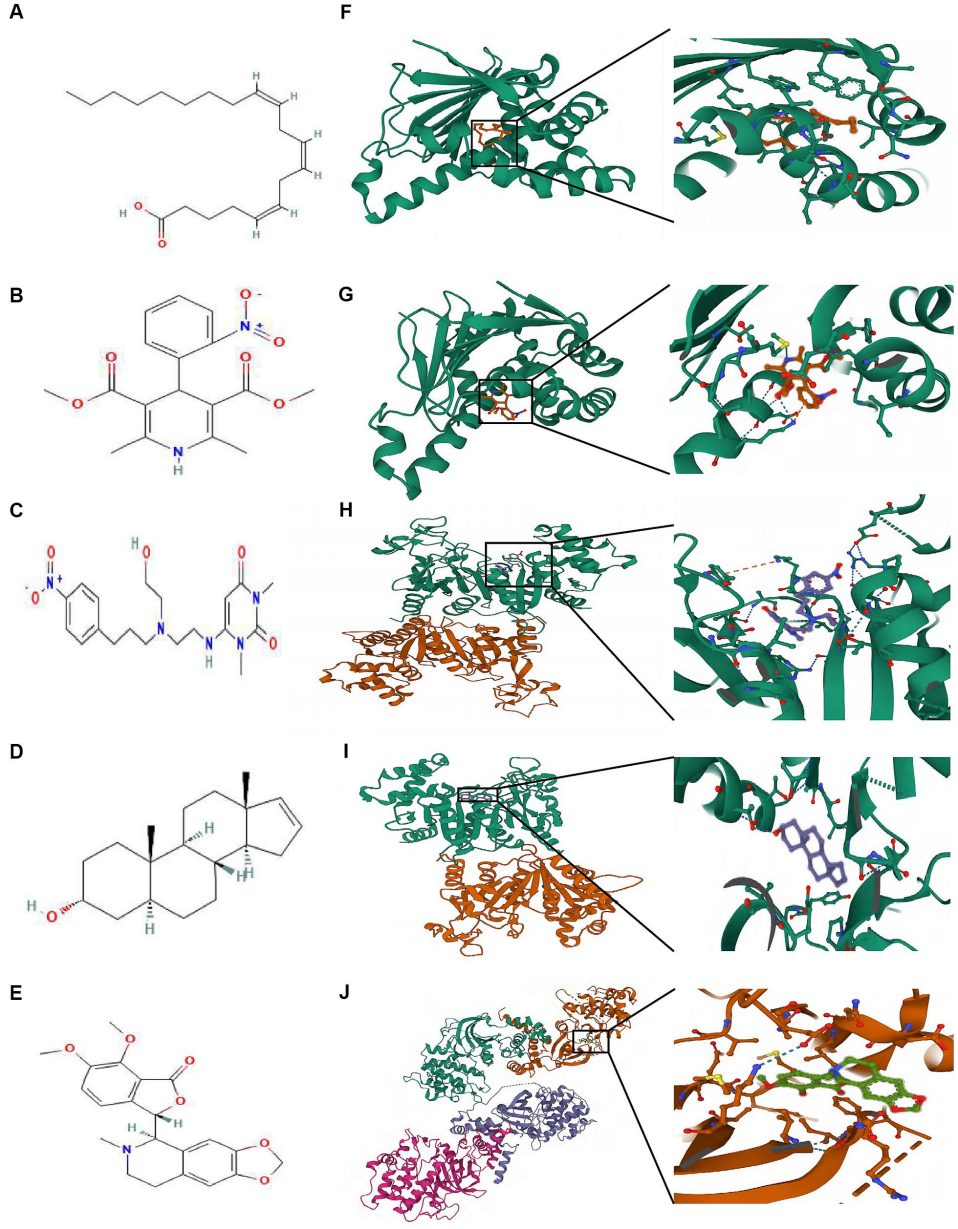
The molecular docking method of binding screened drugs to their targets. The 2D chemical structures of 5 small molecule drugs. **(A)** mead-acid **(B)** nifedipine **(C)** nifekalant **(D)** androstenol **(E)** hydrastine. Molecular docking diagram of small molecule drugs and target genes. **(F)** mead-acid – TRPM2 **(G)** nifedipine – HSP90AA1 **(H)** nifekalant – TRPM2 **(I)** androstenol – TRPM2 **(J)** hydrastine – ROCK1.

The highest negative score was assigned to the top 5 small molecule compounds (mead-acid, nifedipine, nifekalant, androstenol, hydrastine) shown in Table 1, indicating their potential as drugs for ALS. Simultaneously, the 2D chemical structures of these compounds were obtained from PubChem (Figures 7A–E). Subsequently, to assess the binding affinity of the drug candidates with their respective targets, a molecular docking analysis was conducted. Using the Autodock Vina v.1.2.2 program, we employed five drug candidates to acquire the binding poses and interactions with four proteins. Accordingly, each interaction’s binding energy was calculated (Figure 7). Consequently, it was disclosed that the drug contenders displayed noticeable hydrogen bonding and robust electrostatic interactions with their protein targets, simultaneously effectively occupying the hydrophobic cavities of every target. Distinct colors are assigned to individual atoms, with hydrogen represented by white, carbon by green, oxygen by red, and nitrogen by blue. Furthermore, the visualization of the lowest binding energy between the small molecule compounds and the core target was conducted (Figures 7B,F–J). Among them, mead-acid exhibited a low binding energy of −5.127 kcal/mol for TRPM2, while nifedipine showed a low binding energy of −5.63 kcal/mol for HSP90AA1. Additionally, TRPM2 displayed low binding energies of −6.782 kcal/mol and − 7.101 kcal/mol for nifekalant and androstenol, respectively. Furthermore, ROCK1 demonstrated a highly stable binding with hydrastine, as indicated by its low binding energy of −7.743 kcal/mol.

**Table 1:** The top 5 compounds from Cmap’s database with the highest negative enrichment scores.

Rank	Score	Compound	Description
1	−99.82	mead-acid	KPL-1 tumor suppressor
2	−99.82	nifedipine	Calcium channel blocker
3	−99.82	nifekalant	Potassium channel blocker
4	−99.75	androstenol	GABA receptor modulator
5	−99.75	hydrastine	Tyrosine hydroxylase inhibitor

## 4. Discussion

ALS is a progressive neurodegenerative disease characterized by motor neuron degeneration (Schoser et al., 2001). Although the exact pathogenesis remains uncertain, emerging evidence suggests that the immune response plays crucial roles in the development and progression of ALS (Zhao et al., 2013; Jara et al., 2017; Wosiski-Kuhn et al., 2019; Passaro et al., 2021; Rodrigues Lima-Junior et al., 2021). DAMPs are endogenous molecules released in response to cellular damage or death (Zhang et al., 2010), and activate pattern recognition receptors (PRRs) in the immune system (Teissier and Boulanger, 2019). Accordingly, DAMPs may hold valuable implications for predicting disease outcomes in ALS and their presence.

Hence, our study mainly explored the role of DAMPs in ALS and delved into it from the perspectives of single cell and transcriptome. Furthermore, we developed a novel, validated, and precise prognostic framework for ALS while examining the clinical significance and immune cell infiltration patterns in both high and low-risk cohorts. In addition, we utilized molecular docking technology to forecast potential drug candidates, thereby offering a theoretical foundation for the advancement of ALS medications.

From the perspective of single cells, first, 10 cell types were identified through quality control and dimensionality reduction clustering. A heatmap revealed that the expression of DAMP-related genes was predominantly observed in microglia. Analysis of KEGG enrichment results indicated a high association between microglial cell genes and DAMP-associated pathways, providing initial evidence that dying microglia in ALS may contribute to the progression of ALS through DAMPs. Secondly, we performed cell–cell chat analysis to identify 12 ligand-receptor pairs in DAMP-related pathways, wherein microglia acted as ligands. The pair SPP1-CD44 exhibited the highest contribution, further validating the predominant occurrence of DAMP in microglia in ALS. Thirdly, DAMPs are released from damaged or dying cells to activate the innate immune system. Notably, microglia serve as intrinsic immune cells within the central nervous system. Hence, by exploring the considerable heterogeneity of microglia, we identified distinct differentiation fates of microglial cells states using developmental trajectory analysis. Through the use of GSEA, we discovered that this pattern of differentiation is closely associated with neuro-immune biology.

Initially, in terms of bulk RNA-seq, four genes (TRPM2, ROCK1, HSP90AA1, HSPA4) were recognized as prognostic DEGs (*p* < 0.05) following the analysis of univariate and multivariate Cox regression, and LASSO regression. In addition, a predictive risk model was developed based on the expression levels of four reference genes. Afterward, the individuals were categorized into groups of low and high risk depending on their overall survival. Furthermore, we also discovered that the developed prognostic model exhibited autonomous predictive capability in forecasting the overall survival (OS) of individuals with ALS. To confirm the predictive capability, an additional validation dataset, GSE112681, was employed, and similar outcomes were noted in both GSE112676 and GSE112680. Further investigation of the potential underlying mechanism was prompted by the robust predictive ability of the prognostic model. Furthermore, we examined the variation in immune cell infiltration between ALS patients at high risk and low risk, taking into account the influence of the immune microenvironment on ALS prognosis. The high-risk group showed increased expression of activated B cells, activated CD4 T cells, activated CD8 T cells, effector memory CD4 T cells, gamma delta T cells, immature B cells, effector memory CD8 T cells, regulatory T cells, type 1 T helper cells, eosinophils, and plasmacytoid dendritic cells (*p* < 0.05). On the other hand, the low-risk group exhibited higher expression of central memory CD4 T cells, natural killer T cells, macrophages, MDSC, and neutrophils. In general, the model proved to be appropriate for different detection data and accurately forecasted the prognosis and response to DAMP therapy in patients with ALS, thereby establishing a theoretical foundation for developing personalized treatment strategies.

Moreover, we performed the functional investigation and molecular docking analysis, utilizing the genes (ROCK1, TRPM2, HSP90AA1, HSPA4) incorporated in the predictive model, to suggest a therapeutic strategy capable of altering unfavorable prognosis. To begin with, ROCK1, a serine/threonine kinase, has been widely recognized for its participation in diverse intracellular pathways, notably encompassing apoptosis and inflammation, both of which bear paramount significance in the pathological framework of ALS. Furthermore, Capitanio et al. found that blocking ROCK1 activity in mutant SOD1 mice (a model of familial ALS) delayed disease onset and extended survival, indicating a direct influence of ROCK1 on ALS phenotype (Capitanio et al., 2012). Moreover, The involvement of TRPM2 in the process of microglial activation, a distinctive feature of neuronal inflammation and degeneration observed in ALS, introduces an additional dimension to its conceivable influence on the ALS phenotype. As elucidated in a scholarly investigation conducted by Hermosura, et al., the discerned evidence underscores the role of TRPM2-mediated calcium ion (Ca2+) influx in fostering microglial activation, thereby culminating in a consequential exacerbation of neuroinflammation-associated detrimental effects (Hermosura and Garruto, 2007; Hermosura et al., 2008). Also, HSP90AA1 and HSPA4’s chaperone function, which assists in the correct folding and stabilization of client proteins, becomes dysregulated in the context of ALS, thereby impacting the proteostasis network. This dysregulation not only compromises the proper functioning of motor neurons but also contributes to the aggregation of misfolded proteins, a characteristic hallmark of ALS pathology (Ciechanover and Kwon, 2015; Zuehlke et al., 2015; Serlidaki et al., 2020). Hence, the aforementioned literature has documented numerous instances in which the four genes have been demonstrated to actively contribute to shaping the ALS phenotype. Furthermore, Mead acid, an omega-9 fatty acid, is an indicator of essential fatty acid deficiency when found in high levels in the bloodstream (Retterstøl et al., 1995). Healthy controls (HC) and those with Alzheimer’s disease (AD) had higher levels of mead acid than HC, suggesting the importance of creating new dietary intervention methods to slow down the advancement of the condition (Iuliano et al., 2013). Secondly, nifedipine, known as a calcium channel blocker, markedly showed an ameliorating effect on the motor deficiencies of various motor neuronal degeneration models, specifically in ALS (Ikenaka et al., 2019). Thirdly, nifekalant, a pure potassium channel blocker, is extensively used to treat fatal ventricular tachyarrhythmia (Harayama et al., 2011). Fifthly, hydrastine is an alkaloid compound and possesses various medicinal properties, including anti-inflammatory, antimicrobial, and antiviral effects. Earlier research indicated that the inhibition of GABA(A) receptors in the Lateral Hypothalamic Area (LHA) using hydrastine led to a reduction in both the proportion and reproduction of lymphocytes, thus facilitating cerebellar DAMP modulation (Wang et al., 2011). Hence, these compounds were deemed promising ALS treatment candidates. Nevertheless, additional research is required to determine effective methods of drug delivery to specific targets. Using innovative biomaterials to construct drug delivery scaffolds may be a beneficial choice.

Our investigation exhibited several advantages. The hub genes were double-validated between single-cell transcriptome and microarray datasets. Moreover, the analysis of small molecule drug compounds for ALS involved the utilization of the CMAP database, which is a validated experimental drug database. Additionally, there are various constraints in the current investigation. Future work is needed as our findings are currently in the stages of analysis and speculation and have not yet been experimentally confirmed. Therefore, future research will focus on investigating the collective therapeutic benefits of these five specific medications at both the cellular and animal levels.

## 5. Conclusion

With scRNA-seq as well as bulk RNA-seq, we first examined the gene landscape of DAMPs in ALS. ALS patients were more likely to have DAMPs in their microglial cells. Our prognostic risk model demonstrated independent prognostic value. In addition, our study contributed to a deeper understanding of immune-infiltrating cells as they relate to ALS. The analysis of molecular docking on DAMPs-related genes might offer new insights into potential ALS treatment strategies.

## Data availability statement

The original contributions presented in the study are included in the article/Supplementary material, further inquiries can be directed to the corresponding author.

## Author contributions

YS: Conceptualization, Data curation, Formal analysis, Funding acquisition, Investigation, Methodology, Project administration, Resources, Software, Supervision, Validation, Visualization, Writing – original draft. RZ: Writing – review & editing.

## Footnotes

^1^ https://pubchem.ncbi.nlm.nih.gov/

^2^ http://www.rcsb.org/pdb/home/home.do

^3^ http://autodock.scripps.edu/
