# Supplementary material for: Analysis of damage-associated molecular patterns in amyotrophic lateral sclerosis based on ScRNA-seq and bulk RNA-seq data
Source: Front Neurosci. 2023 Oct 24;17:1259742. doi: 10.3389/fnins.2023.1259742 (PMC10628000; doi:10.3389/fnins.2023.1259742)
Supplement: Supplementary file 4 [file Table_4.DOCX]

Supplementary Material

**Analysis of Damage-associated Molecular Patterns in Amyotrophic Lateral Sclerosis Based on ScRNA-seq and Bulk RNA-seq Data**

Yue Shi, Ruixia Zhu*

*** Correspondence:** Ruixia Zhu : zrx_200626313@163.com

# Supplementary Figures and Tables

Figure S1 Before the removal of the Umap distribution. (B)After the removal of the Umap distribution

Figure S2 A univariate Cox regression analysis of 19 genes.

Figure S3 Dot plots of the 4 gene signatures.

Figure S4 4 gene signatures in microglia of ALS in cell trajectory curve.

Figure S5 Feature plots showing the top5 marker gene expressions across the 10 cell types.

Figure S6 KEGG enrichment pathways associated with DAMPs-related genes.

Figure S7 KEGG analysis of the differentially expressed genes between the Microglia and Oligodendrocyte clusters；Microglia and In clusters；Microglia and Ex clusters；Microglia and Astrocyte clusters；Microglia and Mural clusters；Microglia and Fibroblast clusters；Microglia and Endothelial clusters；Microglia and T_Cell clusters.

Table S1 7877 DEGs between ALS and control.

Table S2 Details of DAMPS related genes included in this study.

Table S3 Marker genes related to Microglia clusters.
