# Supplementary figures and images for: Analysis of damage-associated molecular patterns in amyotrophic lateral sclerosis based on ScRNA-seq and bulk RNA-seq data
Source: Front Neurosci. 2023 Oct 24;17:1259742. doi: 10.3389/fnins.2023.1259742 (PMC10628000; doi:10.3389/fnins.2023.1259742)

A

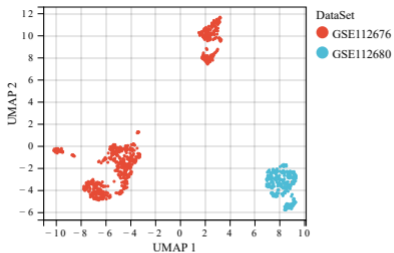

B

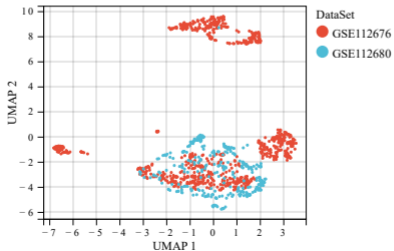

Supplement: Supplementary file 5 [file Data_Sheet_1.PDF]

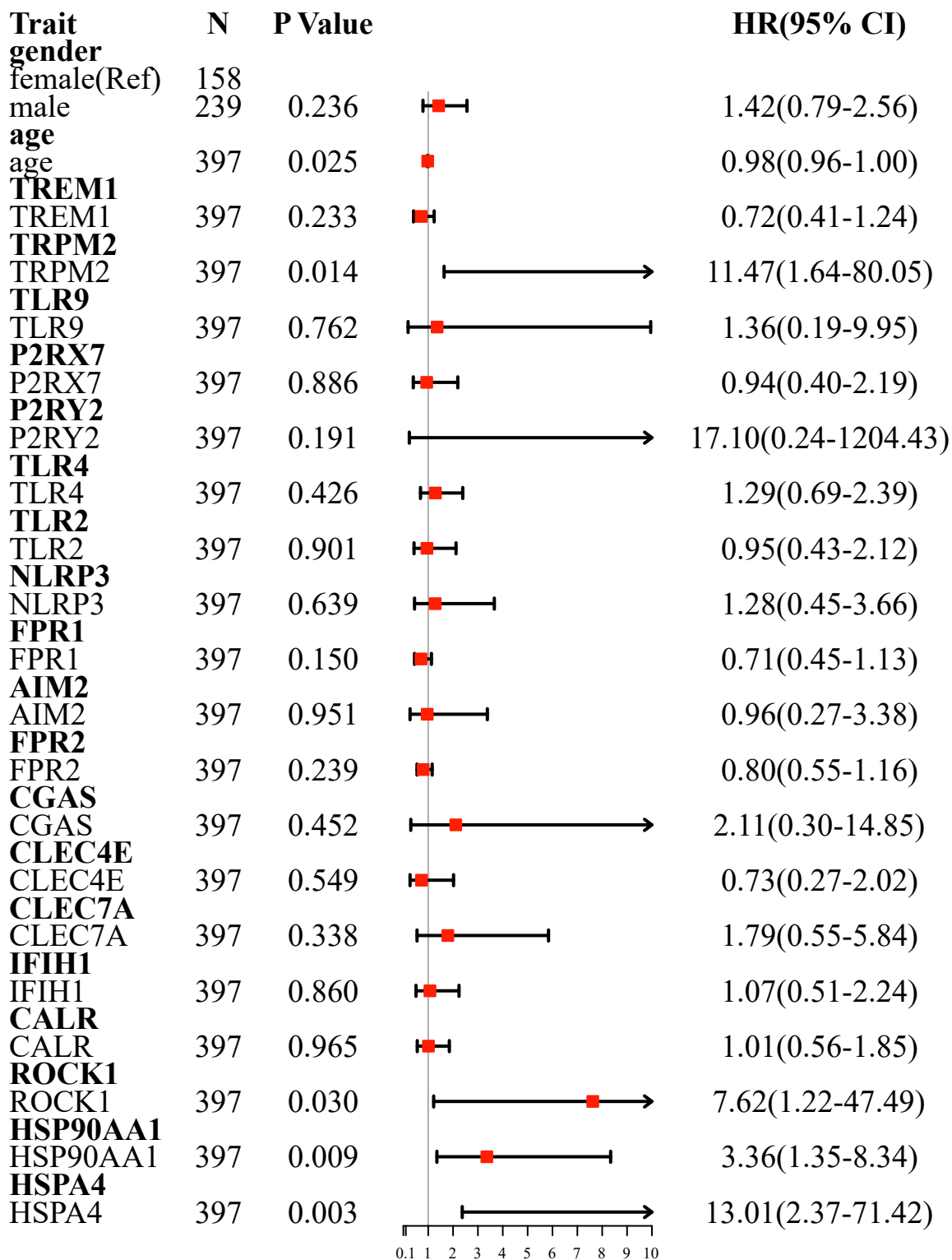

Supplement: Supplementary file 6 [file Data_Sheet_2.PDF]

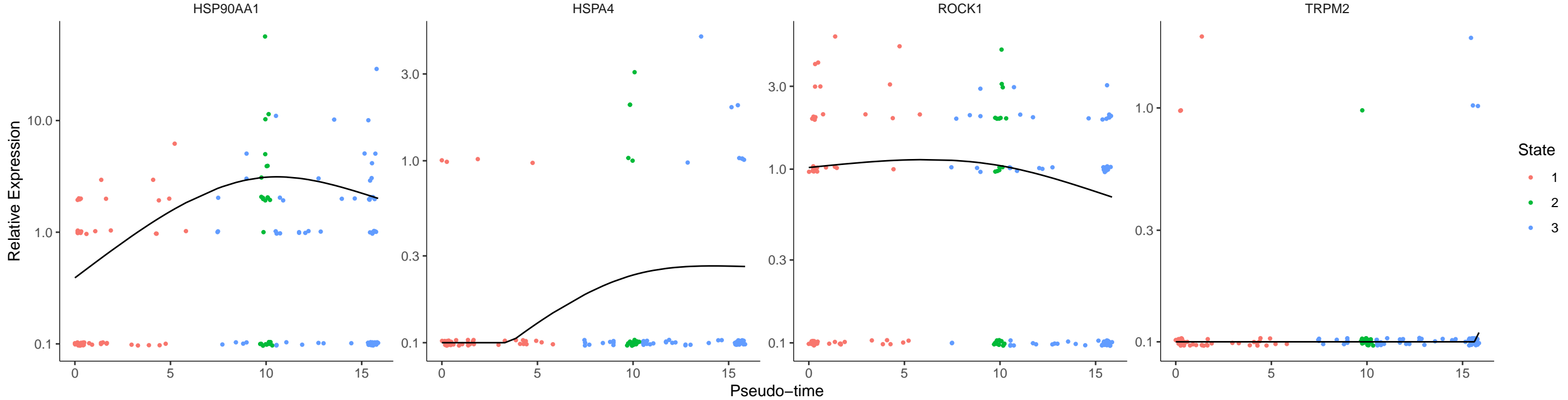

Supplement: Supplementary file 8 [file Data_Sheet_4.PDF]

A

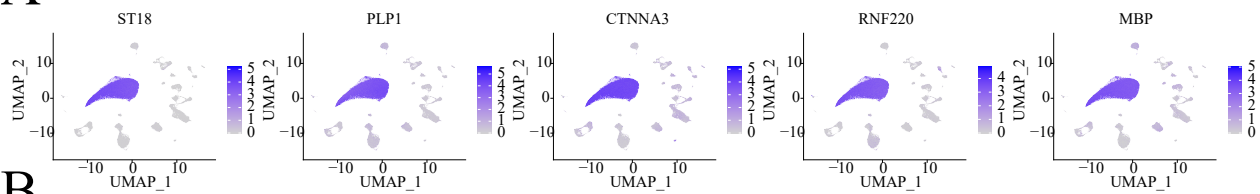

B

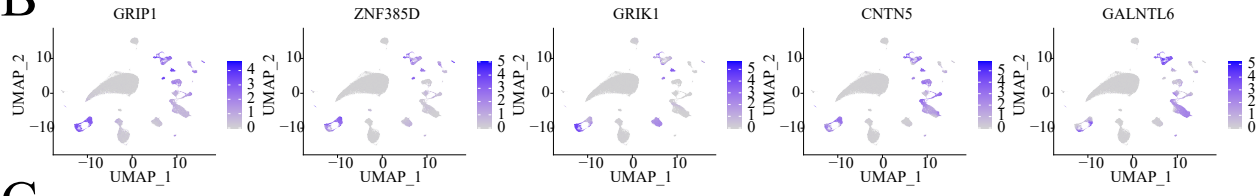

C

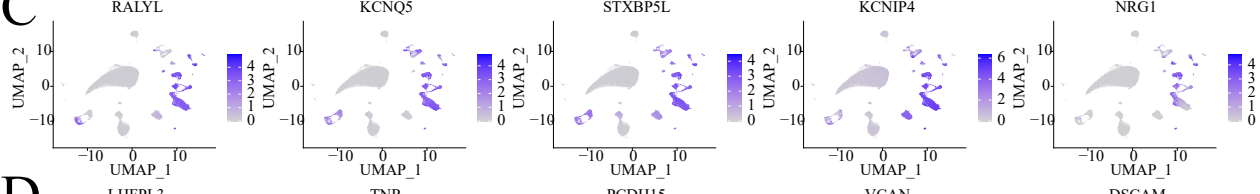

D

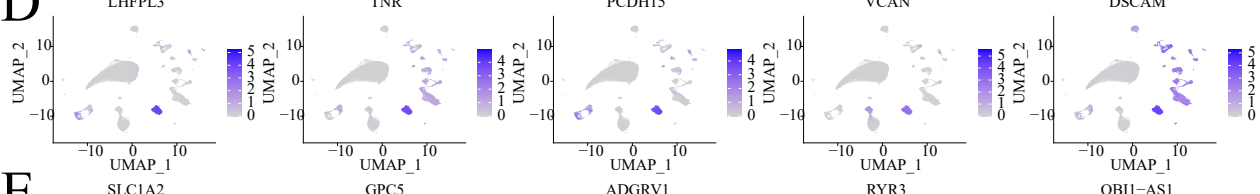

E

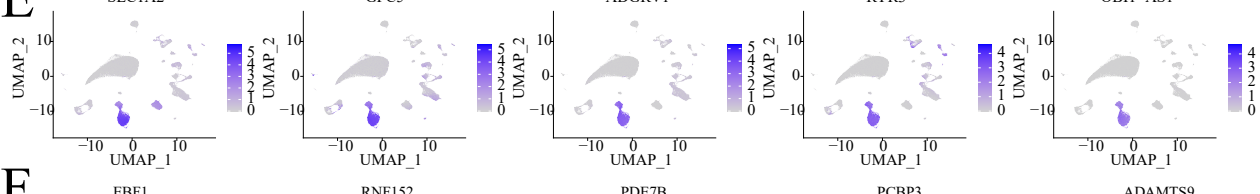

F

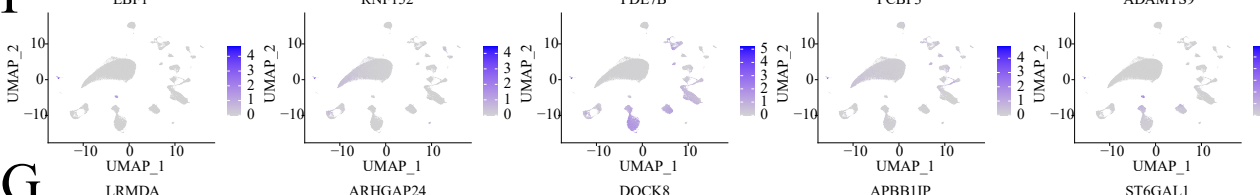

G

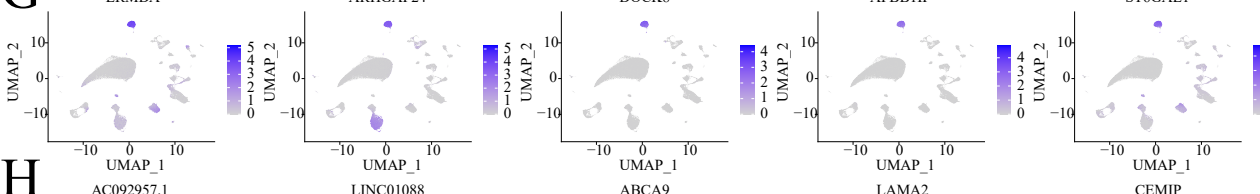

H

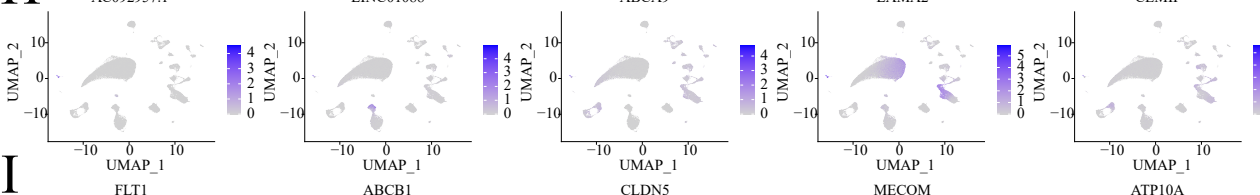

I

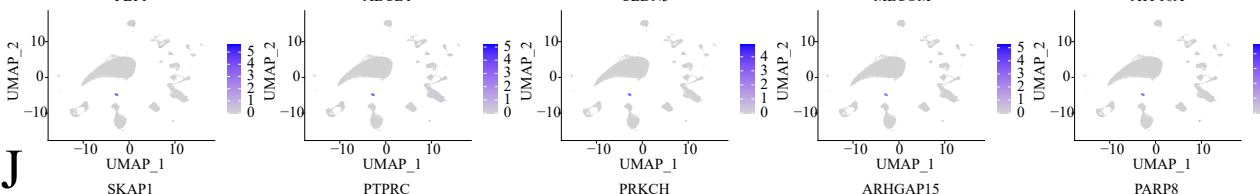

J

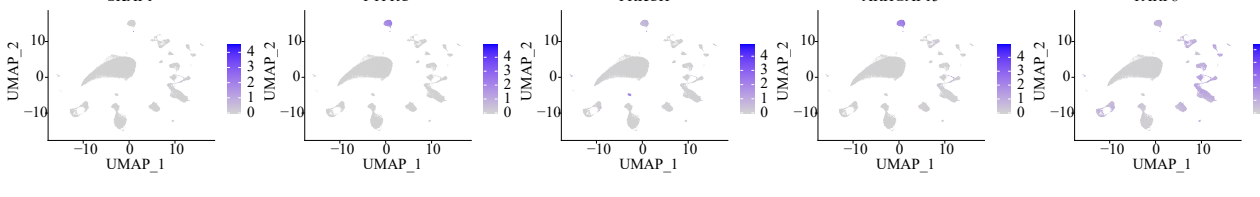

Supplement: Supplementary file 9 [file Data_Sheet_5.PDF]

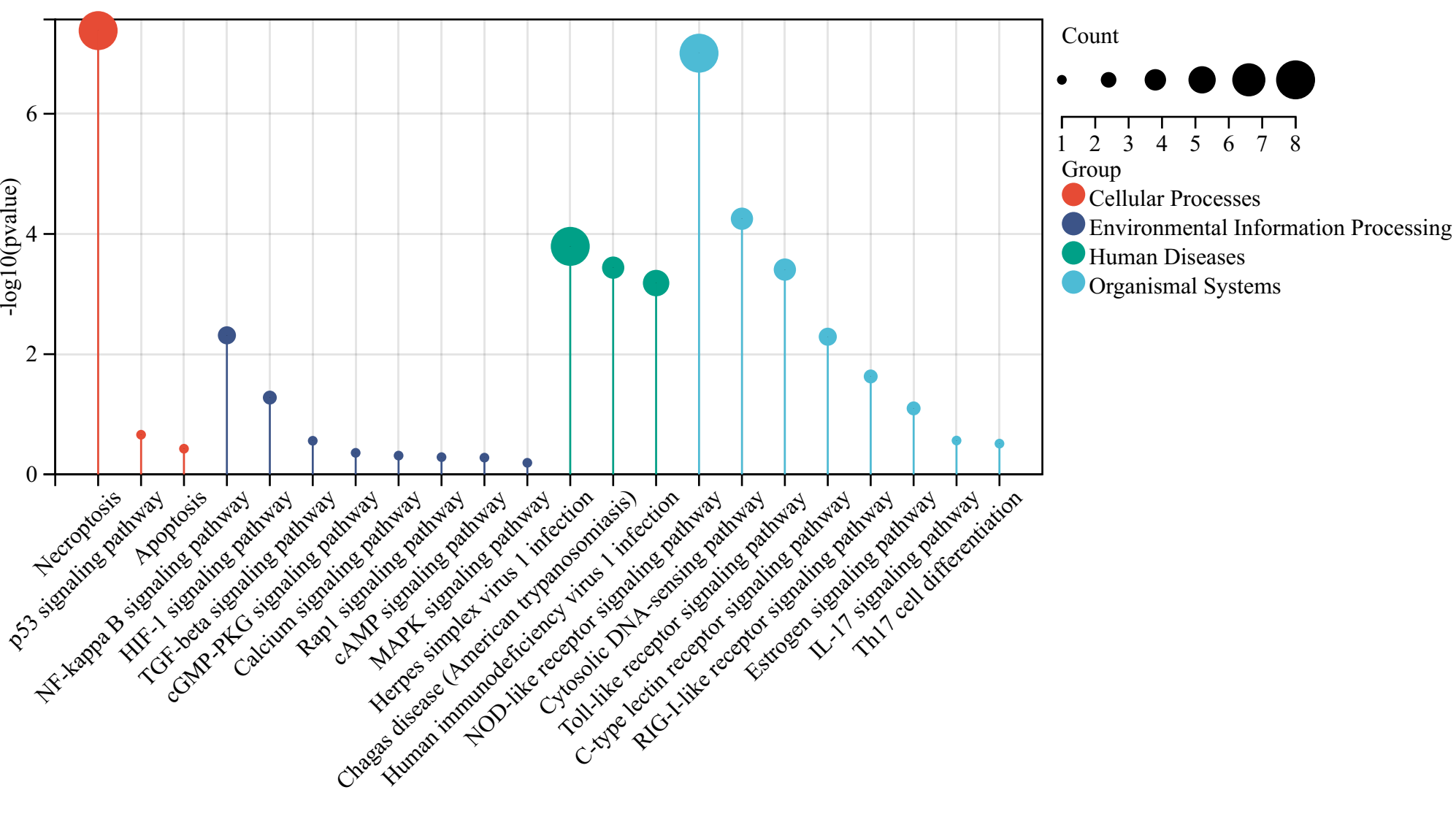

Supplement: Supplementary file 10 [file Data_Sheet_6.PDF]

A

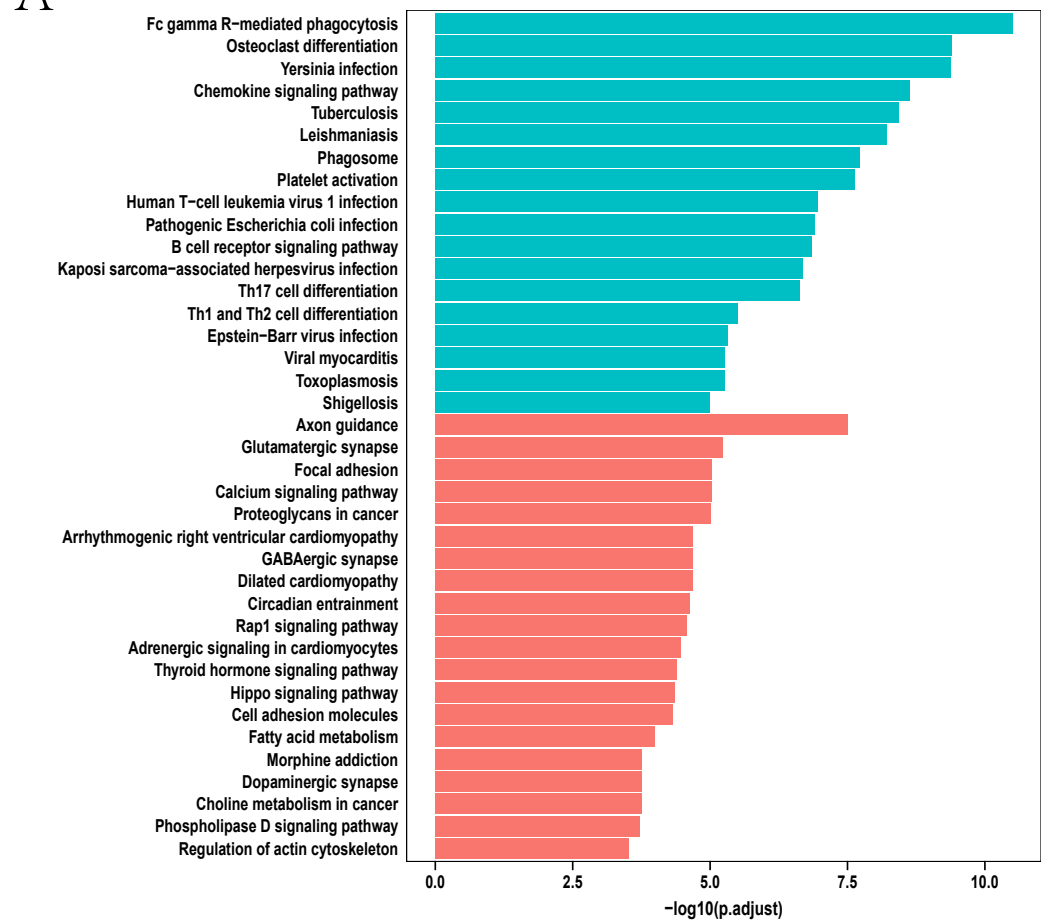

B

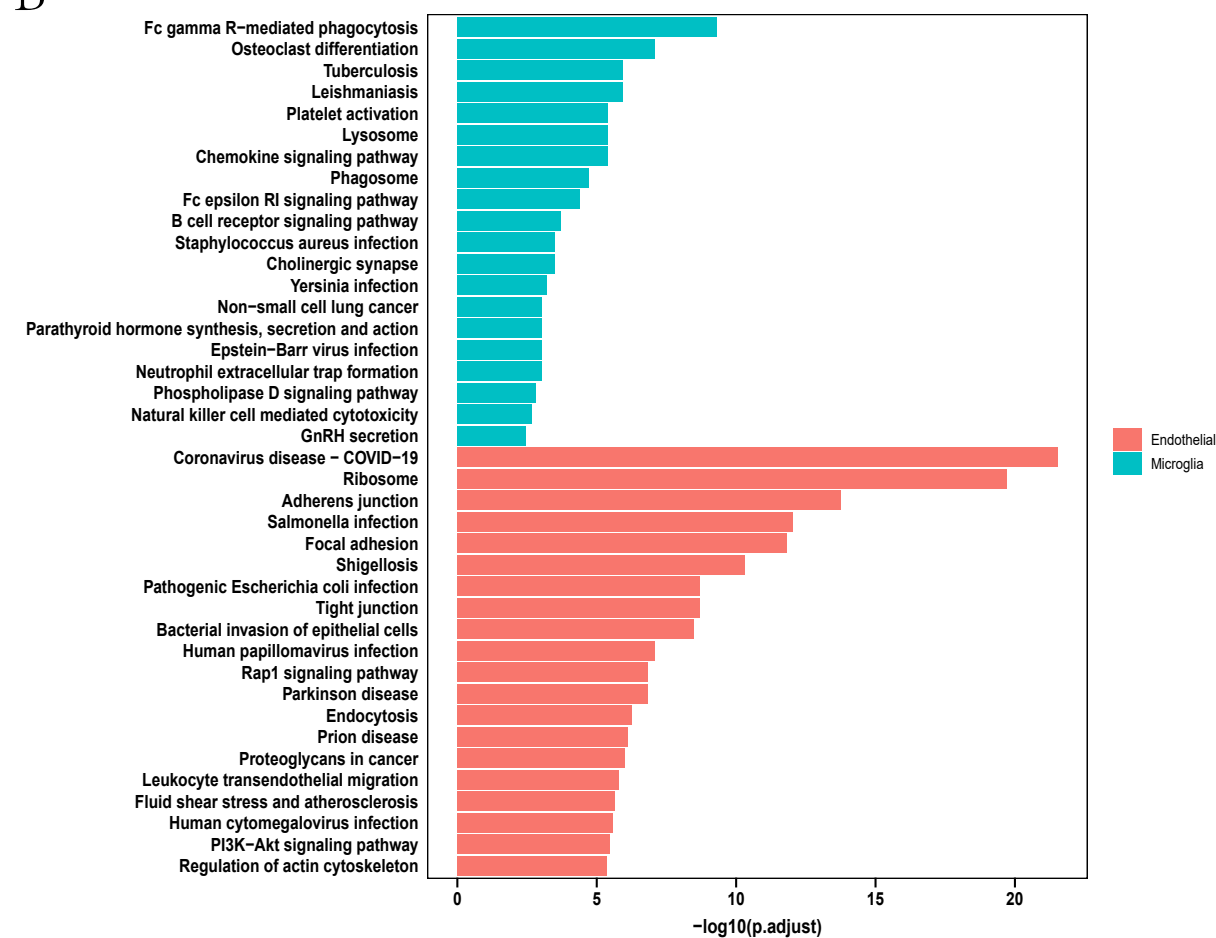

C

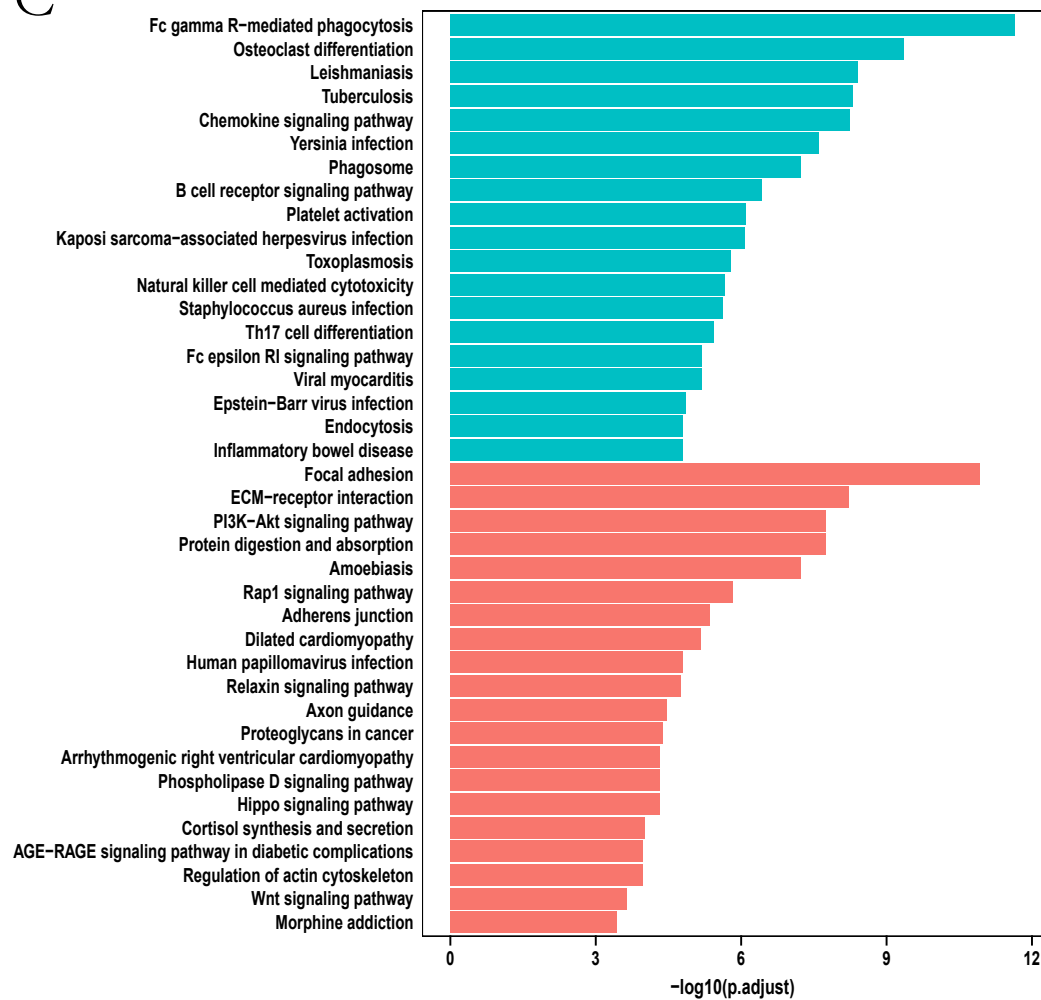

D

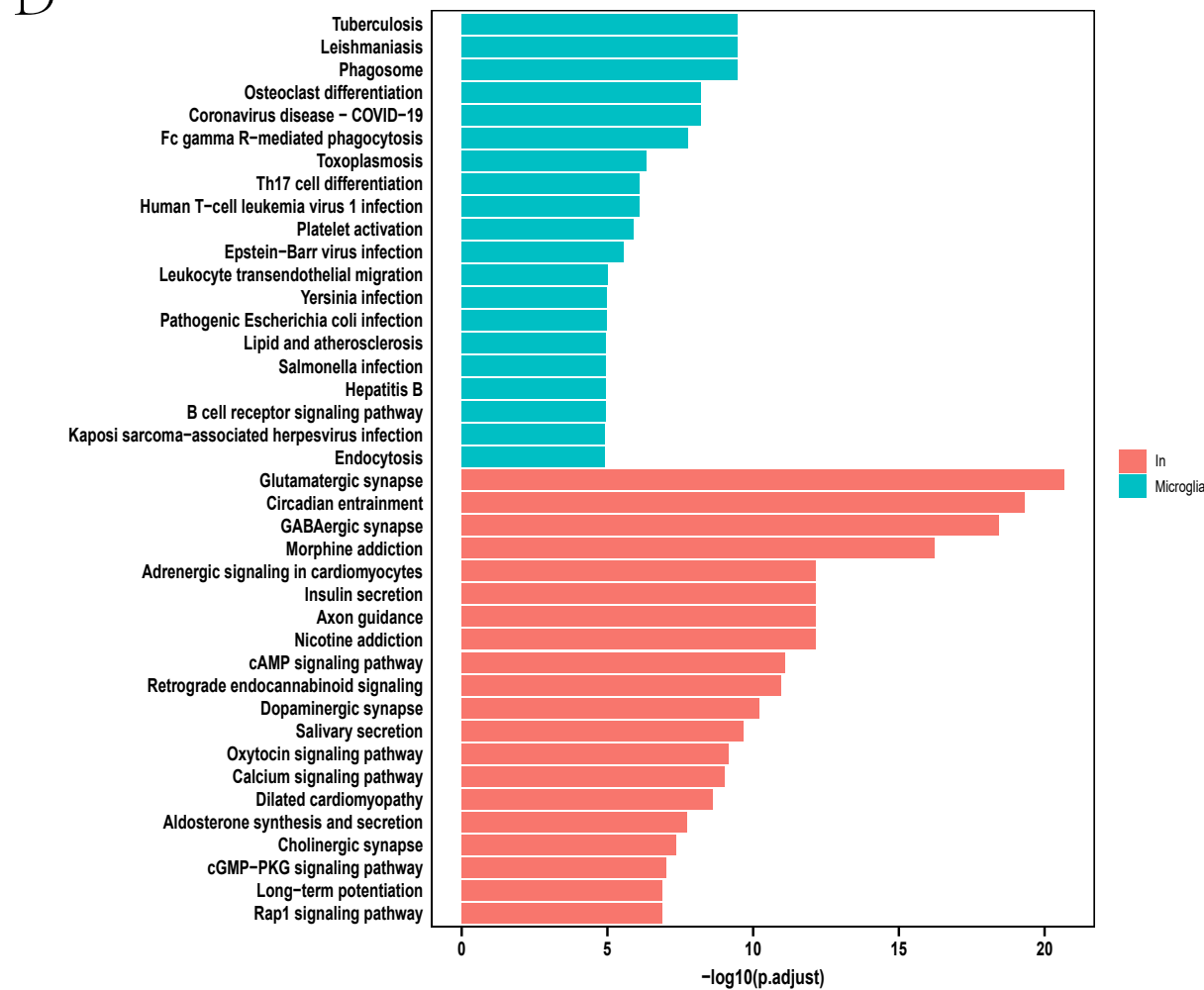

E

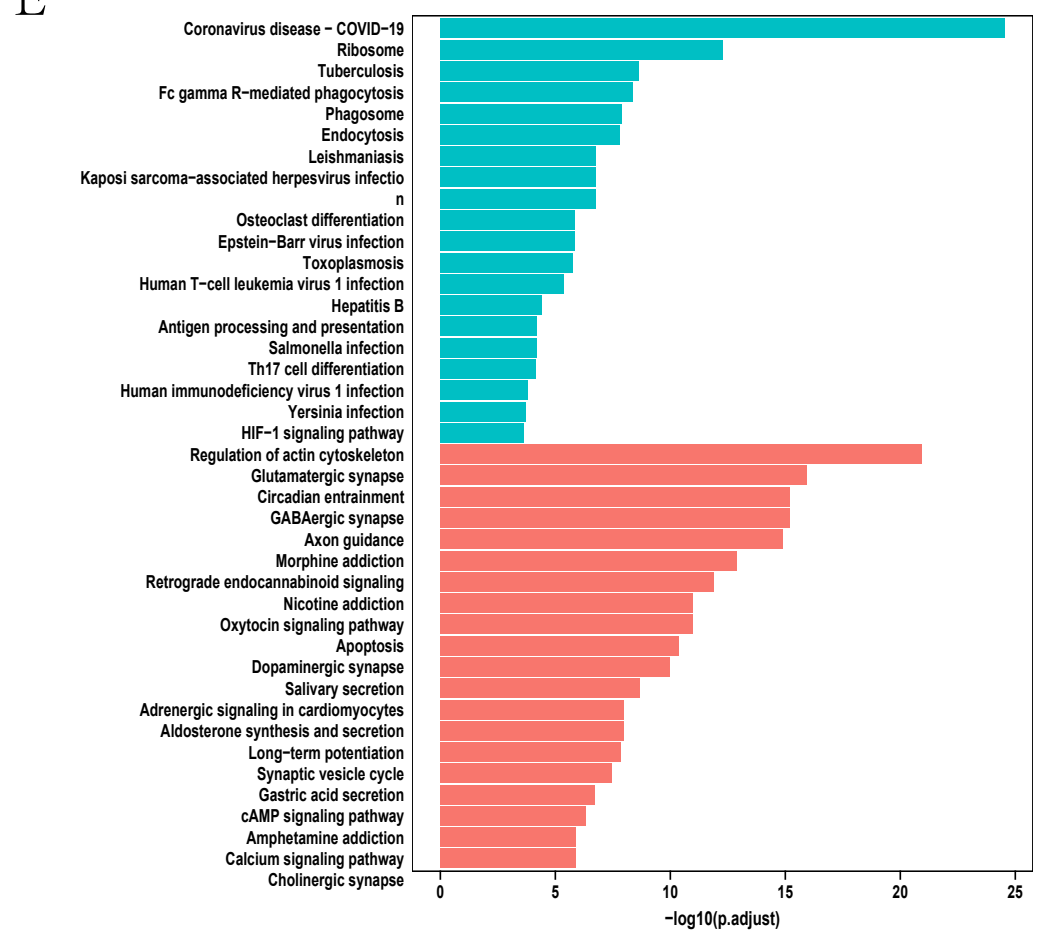

F

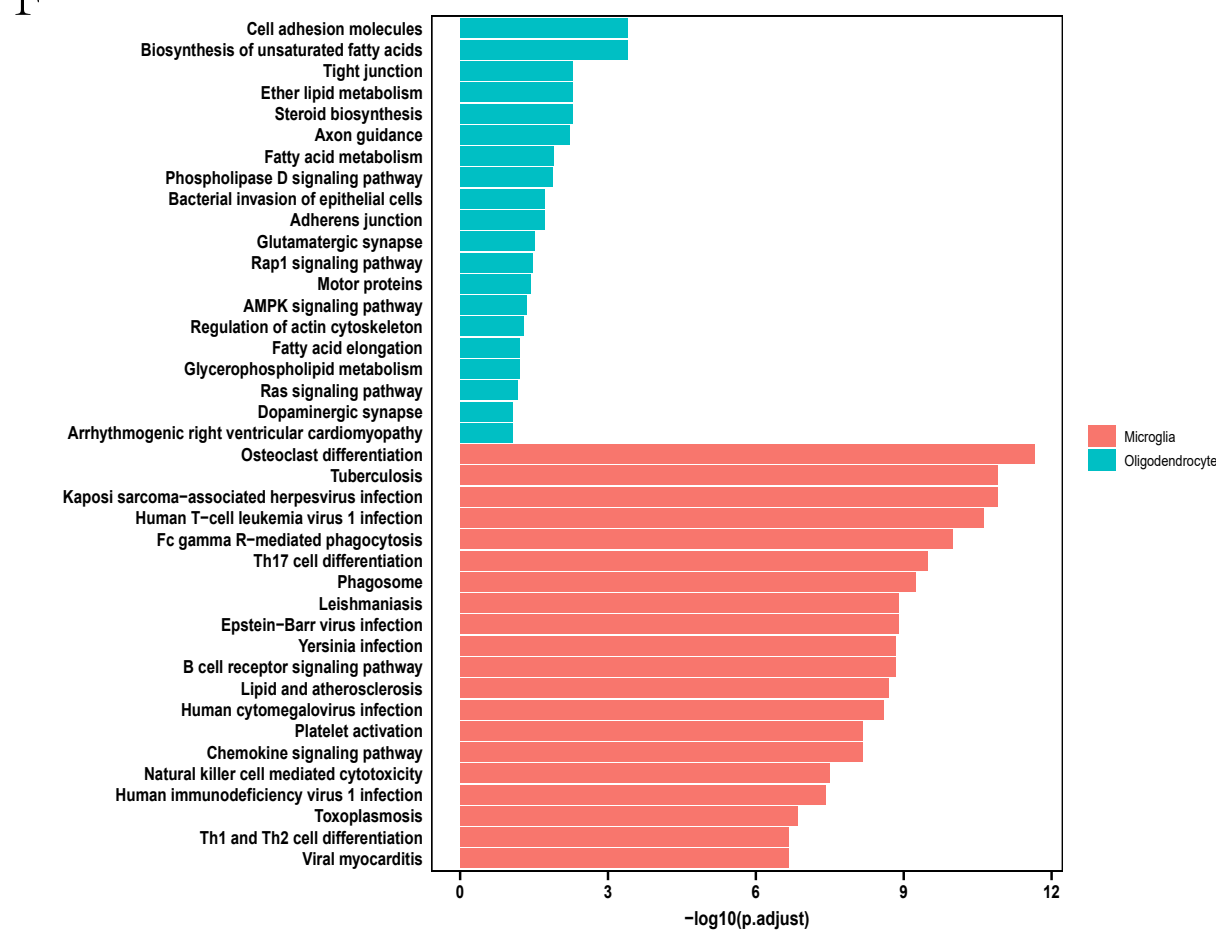

G

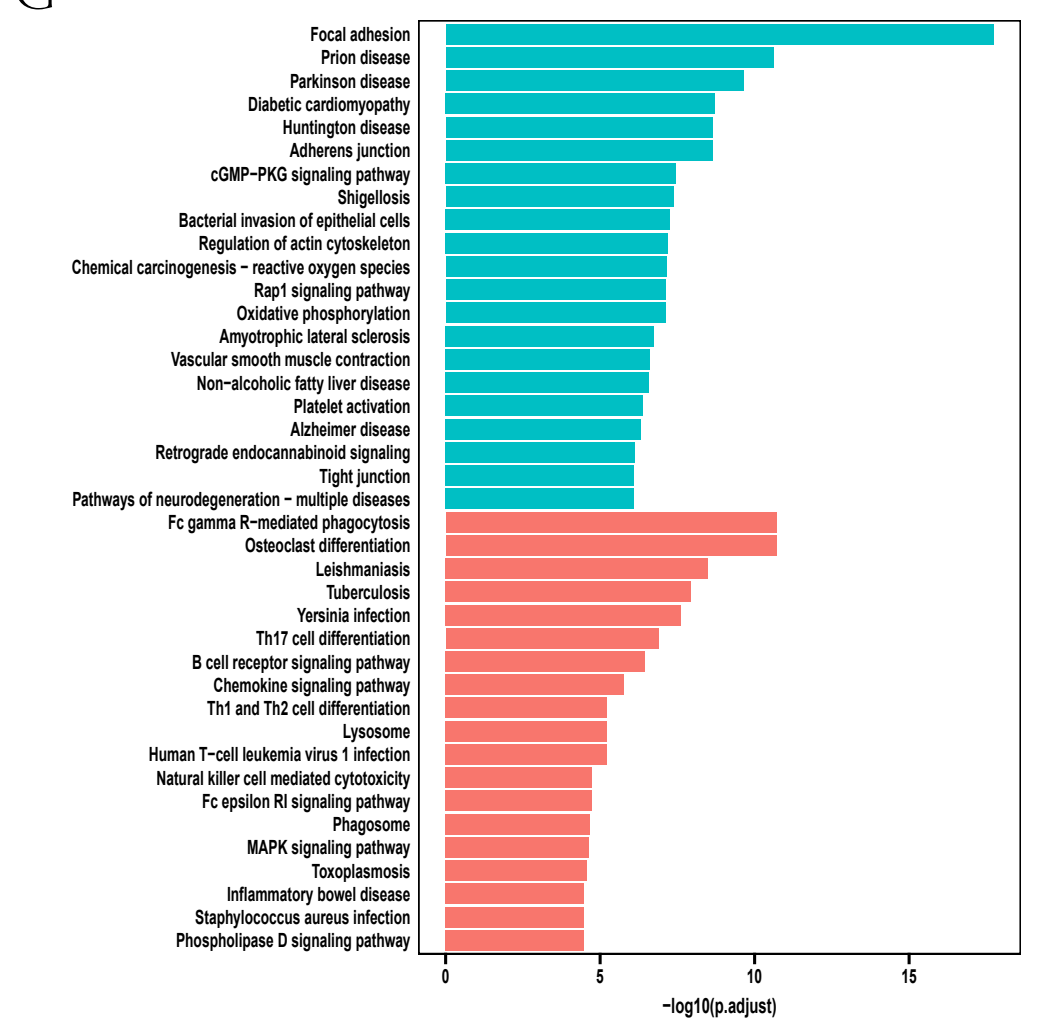

H

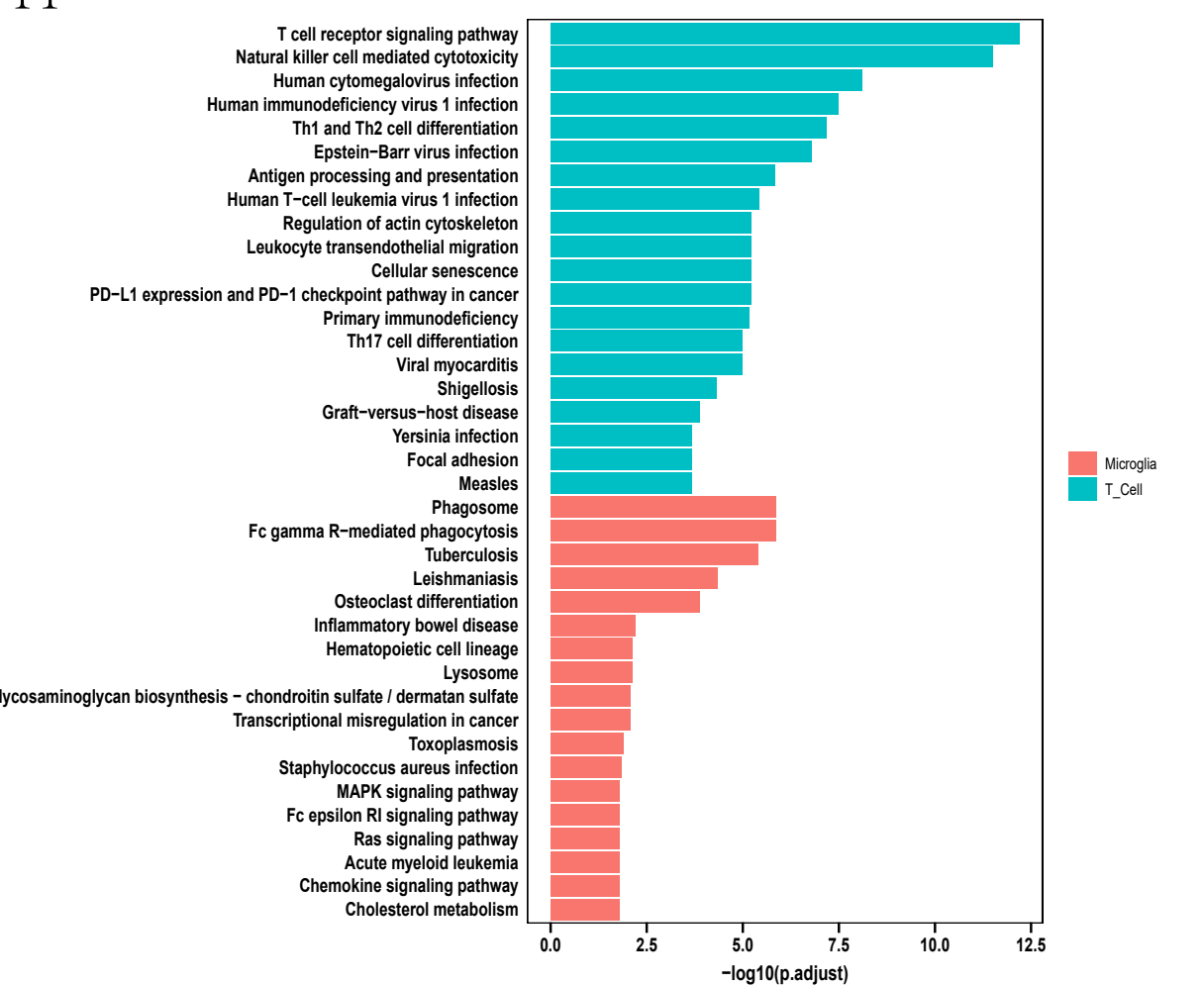

Supplement: Supplementary file 11 [file Data_Sheet_7.PDF]
