# Supplementary material for: Analysis of damage-associated molecular patterns in amyotrophic lateral sclerosis based on ScRNA-seq and bulk RNA-seq data
Source: Front Neurosci. 2023 Oct 24;17:1259742. doi: 10.3389/fnins.2023.1259742 (PMC10628000; doi:10.3389/fnins.2023.1259742)
Supplement: Supplementary file 7 [file Data_Sheet_3.PDF]

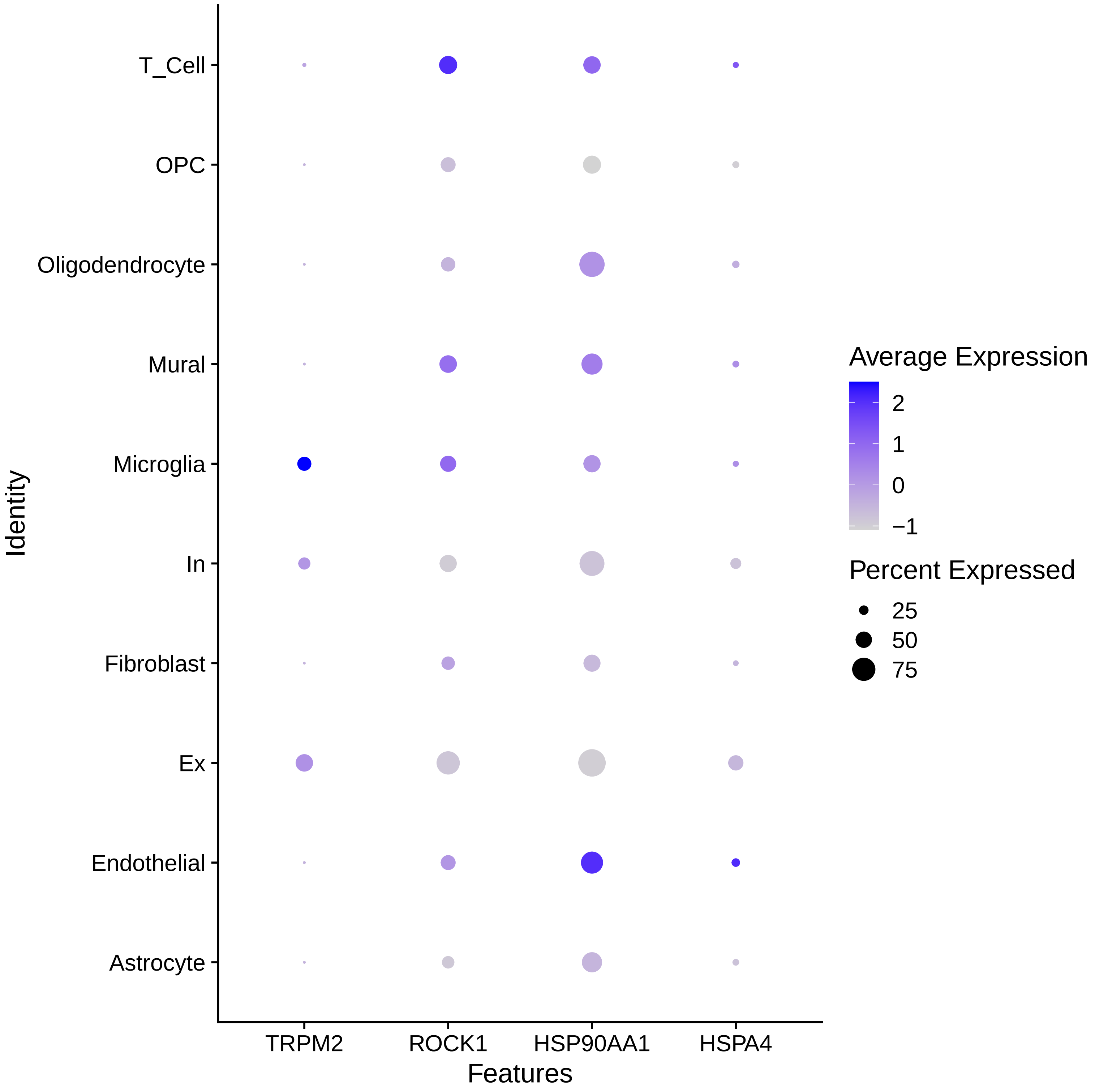

T_Cell
OPC
Oligodendrocyte
Mural
Microglia
In
Fibroblast
Ex
Endothelial
Astrocyte
Identity
TRPM2
ROCK1
HSP90AA1
HSPA4
Features
Average Expression
2
1
0
−1
Percent Expressed
25
50
75
